# Bispecific repurposed medicines targeting the viral and immunological arms of COVID-19

**DOI:** 10.1038/s41598-021-92416-4

**Published:** 2021-06-24

**Authors:** Martin A. Redhead, C. David Owen, Lennart Brewitz, Amelia H. Collette, Petra Lukacik, Claire Strain-Damerell, Sean W. Robinson, Patrick M. Collins, Philipp Schäfer, Mark Swindells, Chris J. Radoux, Iva Navratilova Hopkins, Daren Fearon, Alice Douangamath, Frank von Delft, Tika R. Malla, Laura Vangeel, Thomas Vercruysse, Jan Thibaut, Pieter Leyssen, Tu-Trinh Nguyen, Mitchell Hull, Anthony Tumber, David J. Hallett, Christopher J. Schofield, David I. Stuart, Andrew L. Hopkins, Martin A. Walsh

**Affiliations:** 1 Exscientia, The Schrödinger Building, Oxford Science Park, Oxford, OX4 4GE UK; 2 Diamond Light Source Ltd., Harwell Science and Innovation Campus, Didcot, OX11 0DE UK; 3 Research Complex at Harwell, Harwell Science and Innovation Campus, Didcot, OX11 0FA UK; 4 Division of Structural Biology, Wellcome Centre for Human Genetics, University of Oxford, Oxford, OX3 7BN UK; 5 Instruct-ERIC, Oxford House, Parkway Court, John Smith Drive, Oxford, OX4 2JY UK; 6 Department of Chemistry, Chemistry Research Laboratory,, The Ineos Oxford Institute for Antimicrobial Research, 12 Mansfield Road, Oxford, OX1 3TA UK; 7 KU Leuven Department of Microbiology, Immunology and Transplantation, Rega Institute, 3000 Leuven, Belgium; 8 Calibr, Scripps Research, 11119 N Torrey Pines Road, La Jolla, CA 92037 USA; 9 Structural Genomics Consortium, University of Oxford, Old Road Campus, Roosevelt Drive, Headington, OX3 7DQ UK; 10 Department of Biochemistry, University of Johannesburg, Auckland Park, 2006 South Africa

**Keywords:** High-throughput screening, SARS-CoV-2, X-ray crystallography, Enzyme mechanisms

## Abstract

Effective agents to treat coronavirus infection are urgently required, not only to treat COVID-19, but to prepare for future outbreaks. Repurposed anti-virals such as remdesivir and human anti-inflammatories such as barcitinib have received emergency approval but their overall benefits remain unclear. Vaccines are the most promising prospect for COVID-19, but will need to be redeveloped for any future coronavirus outbreak. Protecting against future outbreaks requires the identification of targets that are conserved between coronavirus strains and amenable to drug discovery. Two such targets are the main protease (M^pro^) and the papain-like protease (PL^pro^) which are essential for the coronavirus replication cycle. We describe the discovery of two non-antiviral therapeutic agents, the caspase-1 inhibitor SDZ 224015 and Tarloxotinib that target M^pro^ and PL^pro^, respectively. These were identified through extensive experimental screens of the drug repurposing ReFRAME library of 12,000 therapeutic agents. The caspase-1 inhibitor SDZ 224015, was found to be a potent irreversible inhibitor of M^pro^ (IC_50_ 30 nM) while Tarloxotinib, a clinical stage epidermal growth factor receptor inhibitor, is a sub micromolar inhibitor of PL^pro^ (IC_50_ 300 nM, K_i_ 200 nM) and is the first reported PL^pro^ inhibitor with drug-like properties. SDZ 224015 and Tarloxotinib have both undergone safety evaluation in humans and hence are candidates for COVID-19 clinical evaluation.

## Introduction

The Coronavirus disease 2019 (COVID-19) pandemic caused by Severe Acute Respiratory Syndrome coronavirus 2 (SARS-CoV-2) is the largest global health emergency to emerge this century^1^. Severely affected patients can display sepsis, through inappropriate recruitment and expansion of the innate immune response and even patients who have cleared the virus may continue to suffer, in part due to fibrotic lesions^2^. To date there are limited direct antiviral medicines approved for treatment of COVID19, thus we aimed to determine whether any molecules previously approved for clinical study could be repurposed for the treatment of COVID19.

Vaccines against COVID-19 are reducing COVID19 outbreaks and mortality^3^, although viral mutations may compromise the longevity of current vaccines^4^ and may not protect against future coronaviral disease. In response to this there are calls to develop a ‘universal vaccine’^5^. Outside of vaccination small molecule inhibitors may play a role suppressing viral proliferation in patients already infected at an early stage in disease and thus reduce overall disease burden^6^ and have so far shown broad spectrum activity against several coronavirus variants^7,8^.

Existing small molecules for treating COVID19 include the anti-viral RNA polymerase inhibitor remdesivir^9^ (originally developed for Hepatitis C Virus) as well as compounds with anti-inflammatory and immunosuppressant effects such as dexamethasone^10^ and baricitinib^11^. Unfortunately, none of these medicines have delivered meaningful benefits to patient populations, execpt dexamethasone which provides benefits in only the most advanced stages of the disease^10^, although combinations of remdesivir and baricitinib show promising results^11^. Thus there is strong motivation for the discovery of new therapeutics not only for the treatment of acute disease. Antivirals offer the potential of prophylaxis, reduction of transmissibility, treatment of unvaccinated patients and suppression of emergent coronaviruses. Considering the lengthy timescales required to develop and approve new therapeutic agents, repurposing of known drugs can potentially reduce the time to develop new treatments.

The SARS-CoV-2 genome encodes small molecule druggable targets including the main protease^12^ (M^pro^) encoded by non-structural protein 5 (nsp5) and the papain-like protease^8^ (PL^pro^) which is part of non-structural protein 3 (nsp3)^13^. As both proteins are essential for viral replication they present attractive targets for drug repurposing efforts. These cysteine proteases are encoded along with the other 14 nsps by the 5′-terminal open reading frame 1a/b (ORF1a/b), which takes up approximately two-thirds of the viral genome. This leads to the expression of the two large replicase polyproteins pp1a and pp1ab. M^pro^ and PL^pro^ are responsible for the proteolytic cleavage of pp1a and pp1ab which consist of nsps 1–11 and 1–16, respectively^14^. M^pro^ cleaves at 11 sites releasing the functional nsps 4–16, while PL^pro^ cleaves at 3 sites releasing nsps 1–3^15^. Additionally, PL^pro^ acts as a deubiquitinase and deISGylase which may modulate the host anti-viral response via suppression of type-I interferon production^16^. Recent developments have seen a rationally designed M^pro^ inhibitor enter clinical testing^17^.

To rapidly identify potential anti–coronavirus therapeutics, we undertook extensive experimental screens of the drug repurposing ReFRAME library, that consists of 12,000 therapeutic agents against M^pro^ and PL^pro^ to identify clinically-viable agents that may be repositioned to treat COVID-19 and future outbreaks. One compound, SDZ 224015, is a potent irreversible inhibitor of M^pro^, whilst a second, Tarloxotinib, is a sub micromolar inhibitor of PL^pro^.

## Results

### ReFRAME library screening and hit triage

The ReFRAME library comprises 12,000 molecules that have been previously approved for clinical investigation in humans, including all currently approved medicines^18^. Despite the overall quality and relevance of this library, it contains some older compounds with properties less attractive for modern drug discovery, such as polyphenol groups, flavonoids and catechols (sennosides)^19^, reactive Michael acceptors (oxantel)^20^, unattractive molecular weights and poor solubility, many of which can also cause assay interference^21^.

**Figure 1:**
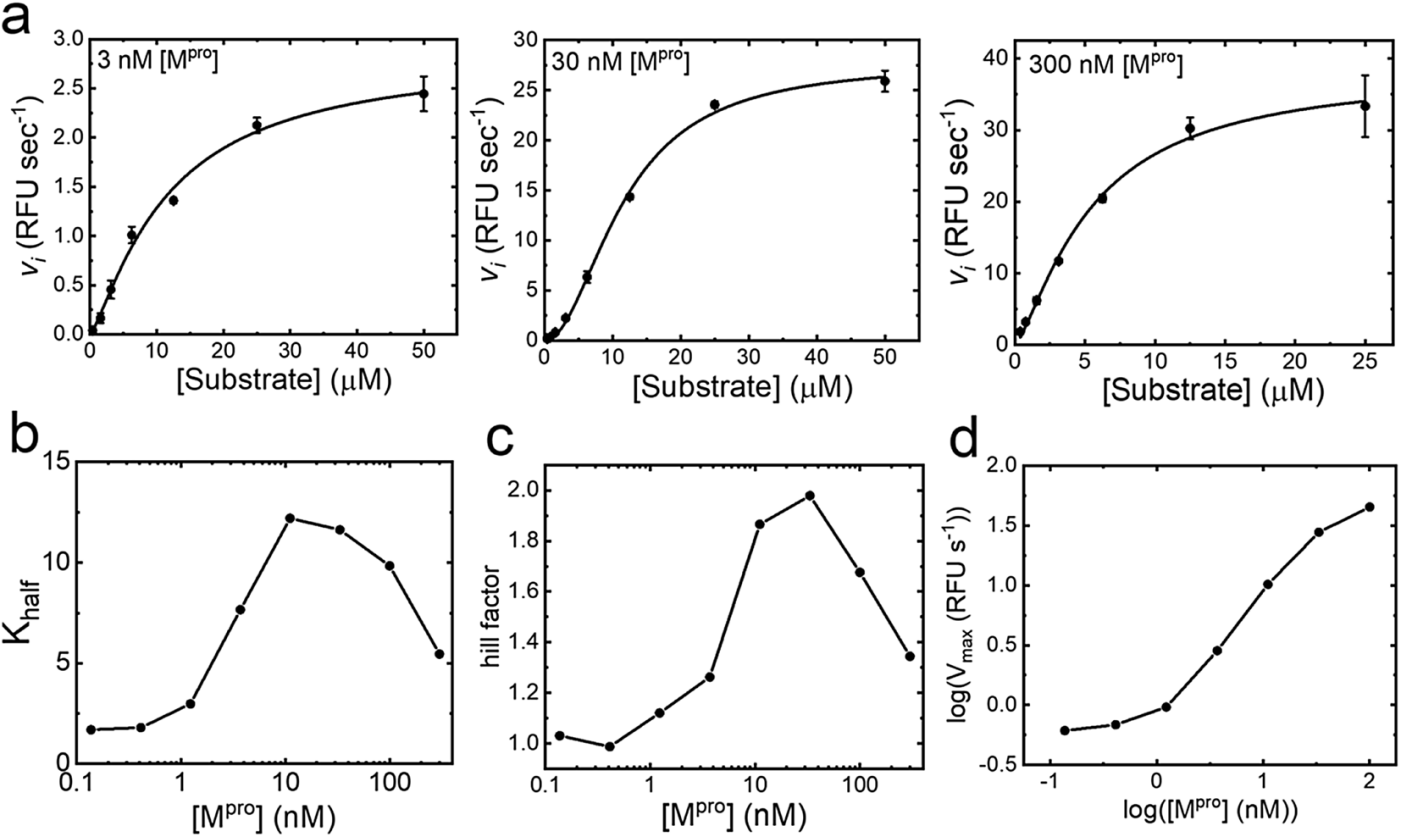
M^pro^ enzymology. Panel (**a**) shows the initial rate of substrate cleavage by M^pro^ at different enzyme concentrations. Data are plotted as an average of four replicates, shown as black circles with error bars representing the standard deviation; a fit of an allosteric sigmoidal model is shown as a black line. Panels (**b**) and (**c**) show a bell-shaped relationship for both the Hill-factor and K_half_ obtained for M^pro^-substrate kinetics at differing concentrations of M^pro^, fitted results are shown as black circles, with lines between the points. Panel (**d**) shows a log–log plot of [M^pro^] vs V_max_; circles show the fitted results.

M^pro^ and PL^pro^ have the potential to exist in several conformationally distinct states, each of which may favour the binding of different inhibitors^22^. M^pro^ substrate velocity titrations revealed evidence for catalytically distinct monomeric and dimeric forms^23^. Dimer dependent catalysis manifests as the observation of enzyme concentration dependent sigmoidal substrate velocity plots (Fig. 1a) as observed for M^pro^ from SARS-CoV-1^24^. The parameters describing the midpoint or slope of the sigmodal substrate velocity plots displayed a bell-shaped relationship with enzyme concertation (Fig. 1b,c), whereas the maximum velocity displayed a sigmoidal relationship with enzyme concentration (Fig. 1d). These results strongly indicate dimerization may be induced either by increasing enzyme or substrate concentration, at high concentrations (> 300 nM) M^pro^ spontaneously dimerizes and low concentration (< 3 nM) M^pro^ behaves as a monomer. These data indicate the potential for inhibitors to bind to these catalytically distinct forms^25^ as well as the potential for binding at the dimer interface^24^. Consequently, the M^pro^ HTS assay was designed to balance these forms.

**Figure 2:**
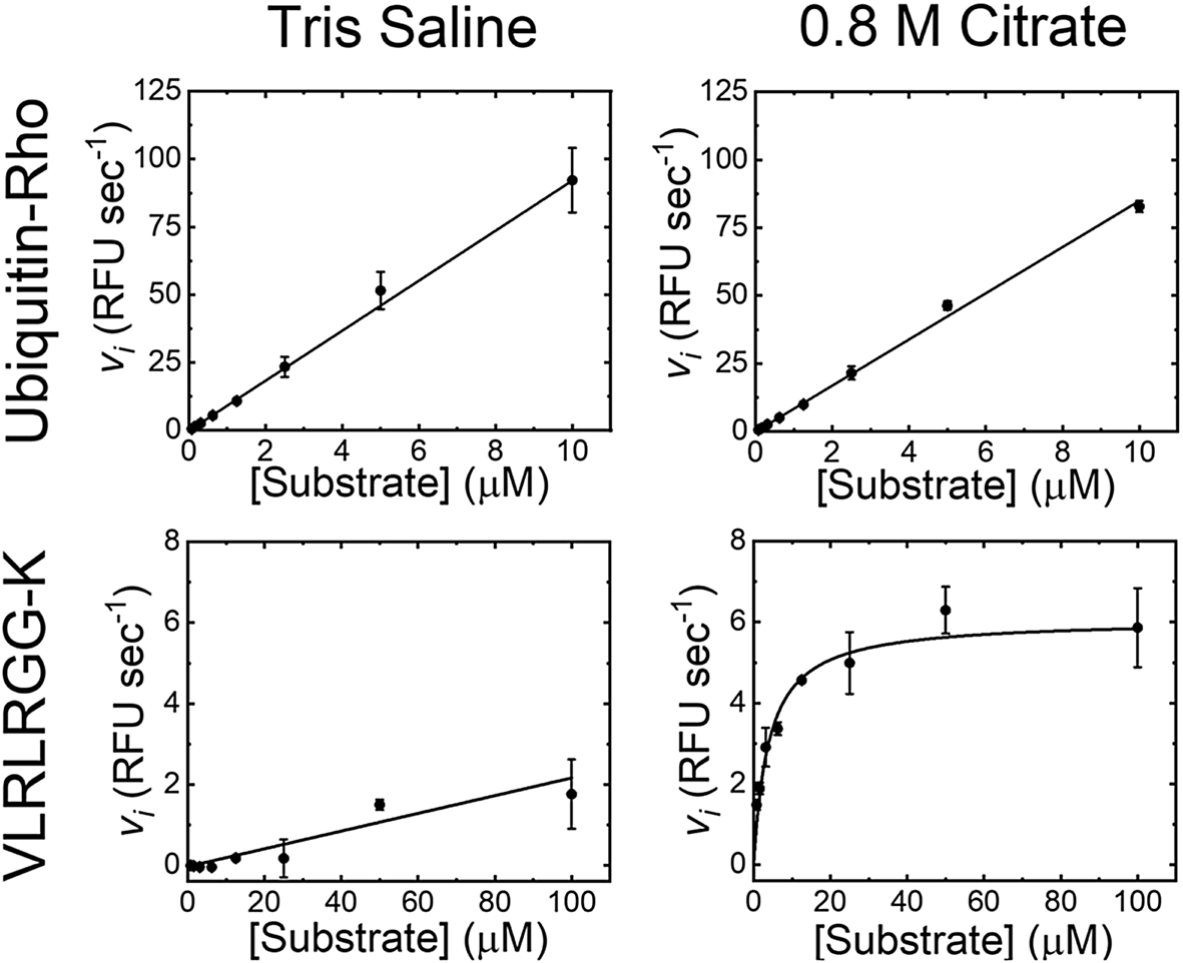
PL^pro^ enzymology. The upper two panels show the initial rate of reaction for ubiquitin-rhodamine cleavage by PL^pro^ in either tris-saline buffer or 0.8 M citrate. The lower two panels show the initial rate of substrate cleavage of a 7-mer peptide corresponding to the C-terminal of ubiquitin in either tris-saline buffer or 0.8 M citrate. The data points show the average of 4-replicates with error bars showing the standard deviation, with either a straight line or Michaelis–Menten fit shown as a solid black line.

PL^pro^ was found to require the presence of high concentrations of anionic Hoffmeister salts to catalyse hydrolysis of small peptide substrates, but not large ubiquitin mimetics (Fig. 2). This indicated that the PL^pro^ active site is not accessible in isotonic buffer, as previously observed for SARS-CoV-1 PL^pro^^26^. This requirement may relate to the subcellular location of nsp3 expressed during viral infection. Nsp3 is expressed on the surface of the endoplasmic reticulum and in combination with nsp4 creates double membraned vesicles^27^. For the papain-like protease of the betacoronavirus murine hepatitis virus, PL^pro^ did not process the viral polyprotein unless expressed on the ER membrane^28^. Thus, to maximise the discovery of inhibitors, the HTS was run in the presence of 0.8 M citrate.

In order to minimise the number of HTS false positives, confirmatory screens were run, followed by a counter screen of M^pro^ and PL^pro^ hits against one another, taking advantage that despite minimal homology both have nucleophilic cysteine containing active sites but recognise distinct peptide substrate sequences.

An initial single point screen of the ReFRAME library performed well for M^pro^ and adequately for PL^pro^ (Supplementary Fig. 1). Following single point screening, a hit triage consisting of repeat confirmation and selectivity counter screening (Supplementary Fig. 2), twenty-one M^pro^ and thirty-five PL^pro^ compounds remained (Supplementary Table 1). After filtering to remove undesirable chemical structures, such as pan assay interference compounds etc., two novel M^pro^ and a single PL^pro^ hit were selected for further analysis.

### SARS-CoV-2 M^pro^ inhibitors

**Figure 3:**
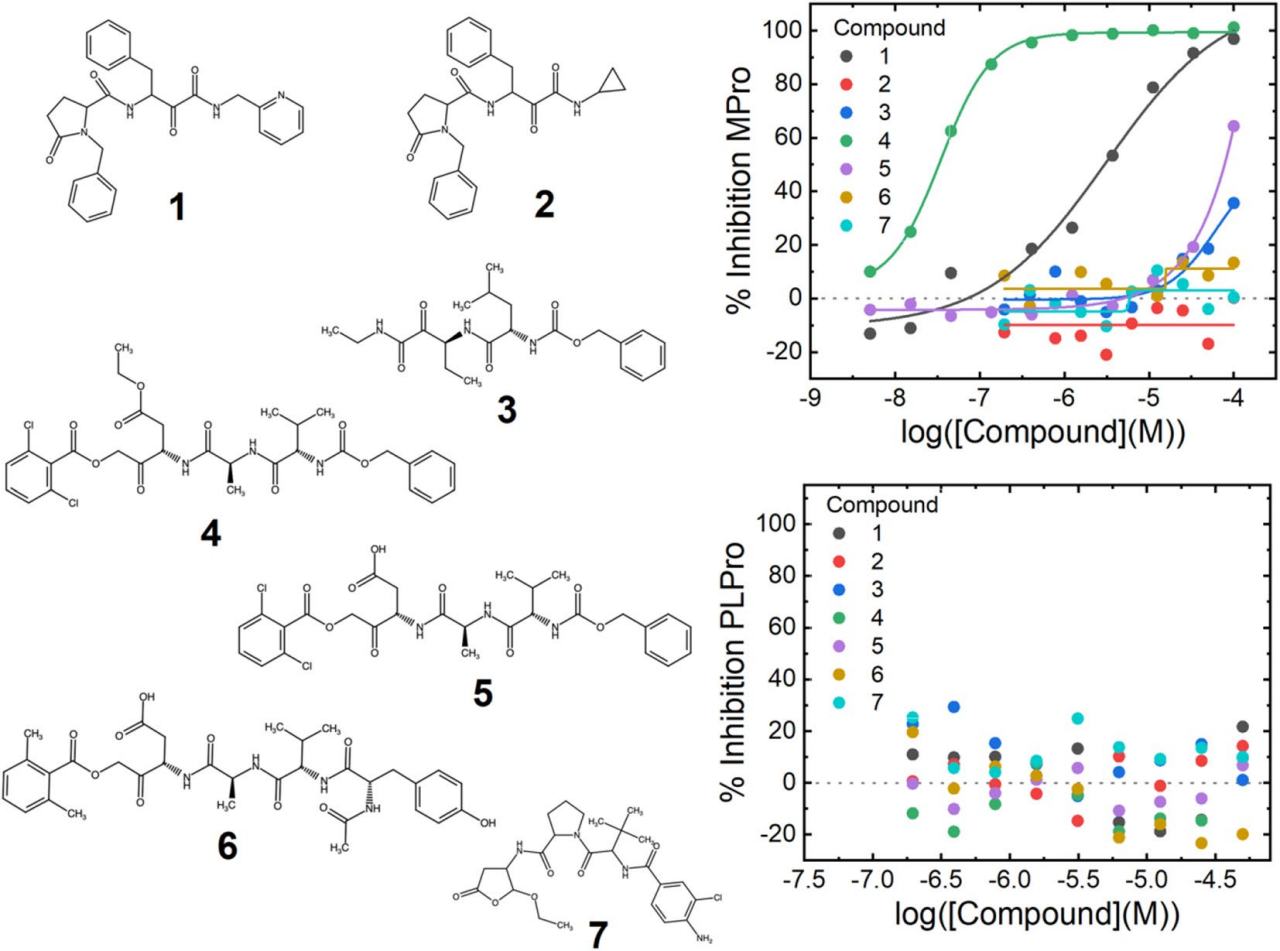
Potent inhibitors of SARS-CoV-2 M^pro^. Structures of the compounds tested against M^pro^ are shown to the left of the graphs. Compounds **1**–**3** are calpain inhibitors and **4**–**7** are caspase-1 inhibitors. **1** is a pyridine analogue of ABT-957 (**2**), **4** is the ester prodrug SDZ 224015 with **5** Caspase-1 active acid version of **4**. The upper graph shows titrations of the compounds plotted against inhibition of M^pro^, and the lower graph shows the same compounds against PL^pro^. Data are singlicate and representative of at least four repeats on separate occasions.

The two selected M^pro^ hits from the ReFRAME screen are compounds **1** and **4** (Fig. 3). **4** has an IC_50_ of 30 nM (the biochemical limit of the assay), however contains a less attractive peptide backbone scaffold, whilst **1** exhibits a weaker IC_50_ of 3 µM and structure consistent with drug-like absorption, distribution, metabolism, and excretion (ADME) properties^29^. **1** is a derivative of the investigational compound ABT-957^30^ (**2**, Fig. 3, Supplementary Table 1) a calpain 1 & 2 inhibitor, differing only by the presence of a pyridinyl group instead of the ABT-957 cyclopropyl-group. This substitution is responsible for the M^pro^ inhibition potency of **1** compared to **2** (IC_50_ > 100 µM, Fig. 3). Inhibition of M^pro^ does not seem to be a general property of calpain inhibitors, as the tool calpain inhibitor Z-L-Abu-CONH-ethyl (**3**, Fig. 3) does not inhibit. **1** was confirmed to bind to M^pro^ in a SPR assay, giving a K_D_ of 1 µM (Supplementary Fig. 3). Furthermore, in HUH7 cells **1** was non cytotoxic at concentrations up to 100 µM and showed an antiviral effect of 75% at 100 µM (Supplementary Fig. 5).

Compound **4** is the investigational caspase 1 inhibitor prodrug SDZ-224015^31^, which is cleaved by esterases in vivo to yield **5**, where the free aspartic acid is revealed (Fig. 3). In contrast to the exquisite potency of **4**, **5** has limited potency for M^pro^, yielding only 50% inhibition at 100 µM. Inhibition of M^pro^ did not seem to be a general property of caspase 1 inhibitors, as the tool tetrapeptide Ac-YVAD-AOM (**6**, Fig. 3) and the investigational caspase 1 drug belnecasan (**7**, Fig. 3) did not substantially inhibit M^pro^. **4** was confirmed to bind to M^pro^ in an SPR assay, although due to the mechanism of action a K_D_ cannot be reported (Supplementary Fig. 3). Compound **4** is a suicide inhibitor which is cleaved by M^pro^, releasing a dichlorobenozic acid leaving group and forming an irreversible covalent adduct by reaction with the nucleophilic cysteine (Supplementary Fig. 4).

**Figure 4:**
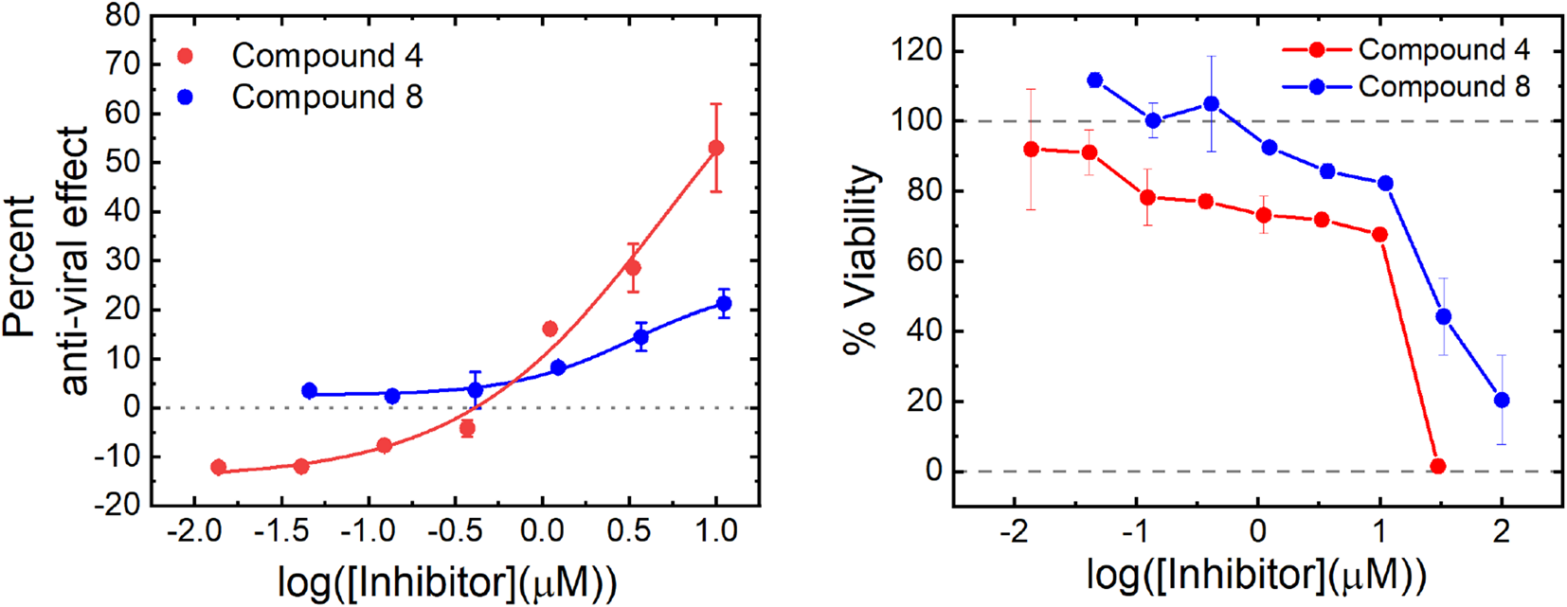
Anti-viral effect of compounds **4** and **8**. The left hand chart shows anti-viral effect of compounds **4** and **8** in HUH7_mCherry cells. An anti-viral effect was established after a 4-day incubation with SARS CoV-2 virus at concentrations which did not cause significant cytotoxic effects. The anti-viral effect was established by an increase in fluorescent cells counted compared to an untreated control. The right-hand chart shows cell viability after 4-days treatment with compounds **4** and **8** in HUH7_mCherry cells. Viability was established by counting the number of fluorescent cells. Data are shown as the average of two technical repeats with error bars representing the range. Data are representative of two technical repeats.

Due to the presence of three esters, **4** is unstable in aqueous media so is unsuitable for the long incubations used in antiviral assays. However, when the dose was refreshed daily, **4** was found to be non-cytotoxic in HUH7 cells at concentrations at or below 10 µM and showed an antiviral effect of 50% at 10 µM (Fig. 4). Refreshing the dose more frequently is likely to further increase the apparent potency.

### SARS CoV-2 PL^pro^ inhibitors

The ReFRAME screen revealed a single inhibitor, tarloxotinib as a potent PL^pro^ inhibitor (Table 1, 8) which has an IC_50_ of 300 nM. **8** is also a prodrug, activated in hypoxic conditions in vivo by STEAP4^32^ to yield the equipotent compound **9** (Table 1) which was designed to target the kinase domain of EGFR^33^. Whilst both **8** and **9** contain a 4-anilinoquinazoline core that is present in several approved drugs^34^, the tested related molecules proved to be less potent than tarloxotinib (Table 1, 10–13).

**Table 1:** Structure–activity relationship of 4-aminoquinazoline EGFR inhibitors against SARS-CoV-2 PL^pro^.


Compound number	Generic name	R^1^	R^2^	R^3^	R^4^	R^5^	IC_50_ (µM) (range)
**8**	Tarloxotinib	**Br**	**F**	**N**	**N**		0.3 (0.1–0.5)
**9**	Tarloxotinib (ac)	**Br**	**F**	**N**	**N**		0.3 (0.1–0.4)
**10**	Pelitinib	**Cl**	**Cl**				> 100*
**11**	Afatinib	**Cl**	**Cl**	**N**			12 (11–16)
**12**	Dacomitinib	**Cl**	**Cl**	**N**			4 (3–5)
**13**	Gefitinib	**Cl**	**Cl**	**N**			7 (6–11)

*The range could not be established due to lack of potency.

Analysis of the other 4-anilinoquinazoline approved medicines revealed the presence of the α,β-unsaturated amide warhead on **8** and **9** was not necessary or sufficient for activity, as **13** achieved weak activity without the warhead whilst the presence of a nitrile on the 3 position of **10** completely removes potency despite the presence of the warhead. These observations show that inhibition is not solely due to intrinsic reactivity of the molecules but requires specific molecular recognition.

**8** was found to be competitive with the peptide substrate (Supplementary Fig. 6) and despite the conditions required for its discovery, the potency of **8** was not dependent on the use of high concentrations of anionic Hoffmeister salts (Supplementary Fig. 7). In HUH7 cells **8** was non-cytotoxic at concentrations up to 10 µM, and showed an antiviral effect of 25% at 10 µM (Fig. 4).

### Crystallography of the ReFRAME hits

**Figure 5:**
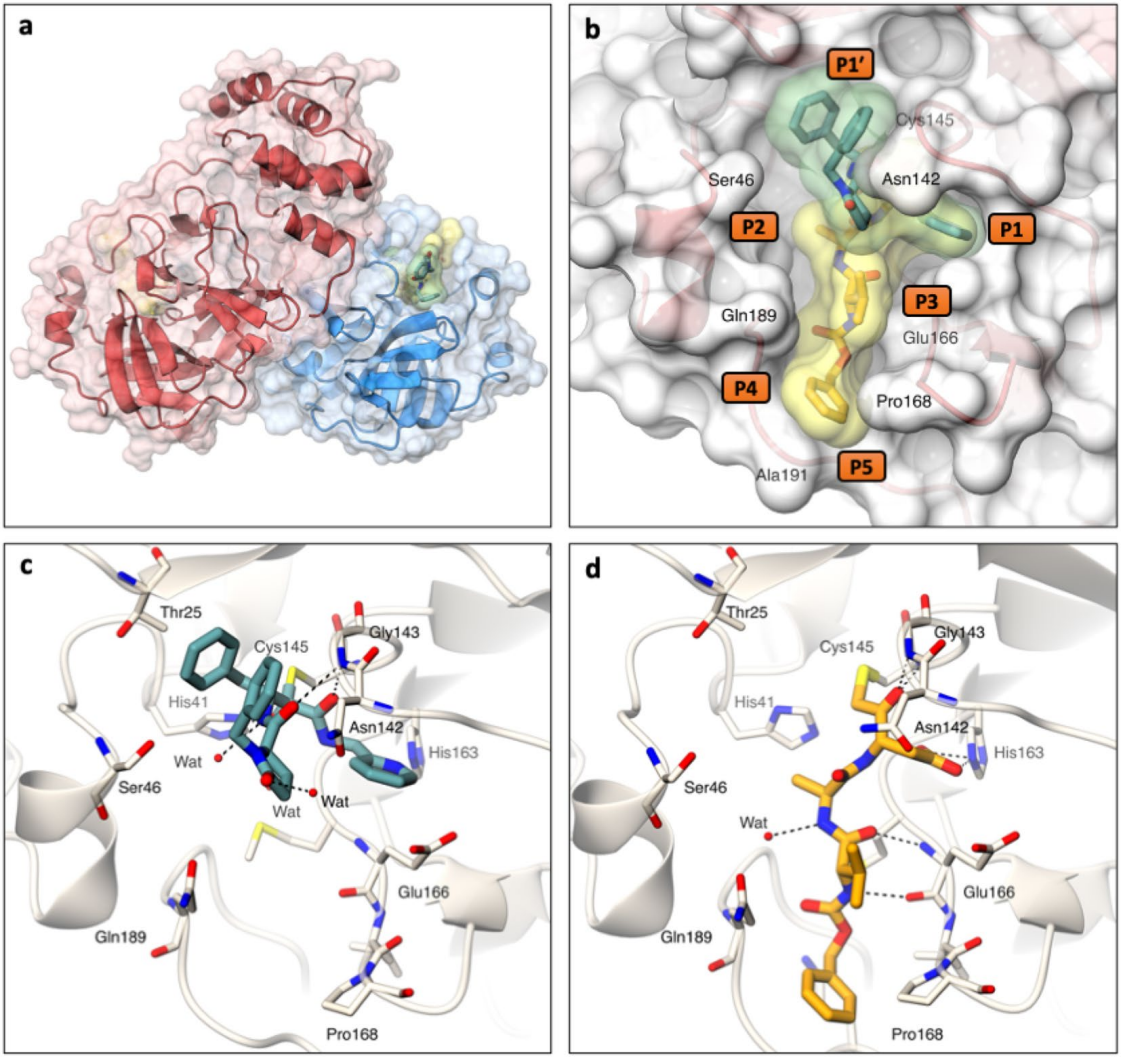
Crystal structures of SARS-CoV-2 M^pro^ in complex with ReFRAME inhibitors. (**a**) Ribbon representation with transparent surface of the M^pro^ dimer coloured in red and blue to delineate each protomer. The structures of M^pro^ in complex with **1** and **5** (sticks with yellow and green transparent surface, respectively) reveal that both bind in the M^pro^ substrate binding pocket. (**b**) Surface representation showing the overall binding modes of compound **1** and **5** (green and yellow transparent surfaces, respectively). (**c**) and (**d**) Stick representations of compounds **1** and **5** showing interactions (hydrogen bonds as dashed lines) within the M^pro^ binding pocket. Structures are deposited in the pdb as 7AEH for **1** and 7AEG for **5**. Figure generated with PyMOL, The PyMOL Molecular Graphics System, Version 2.0 Schrödinger, LLC (https://pymol.org/2/).

X-ray crystal structures were attempted for compounds **1**,**4**,**5** and **8** and obtained for **1** and **5** with M^pro^ (Fig. 5, PDB codes: 7AEH & 7AEG), at 1.3 Å and 1.8 Å resolution, respectively (Supplementary Table 2). The M^pro^ dimer is shown in ribbon representation with **1** and **5** bound at the active site (Fig. 5a,b). Both **1** and **5** bind covalently to the catalytic cysteine (Cys145) with well-defined electron density and form hydrogen bonding networks with the M^pro^ active site (Fig. 5c,d, Supplementary Fig. 8). Despite both **1 **and **5** interacting with Cys145, they have substantially different binding modes. **5 **extends from P1 to P5 of the M^pro^ active site (Fig. 5b). By contrast **1 **binds from P1 to P1′ across the region occupied by the catalytic cysteine, with the two inhibitor benzyl groups π-stacking together to fill the space of the P1′ pocket (Fig. 5b). Electron density for the 2,6-dichlorobenzoate leaving group of **5** was not observed, providing evidence of the proposed mechanism of inhibition (Supplementary Fig. 4 and 8). **1** forms electrostatic interactions with Gly143, Ser144, Cys145, and His41 as well as a water-mediated interaction with His164 whereas **5** makes electrostatic interactions with Gly143, Cys145, His163, His164, and Glu166 together with a water mediated interaction with Gln189 (Fig. 5).

**Figure 6:**
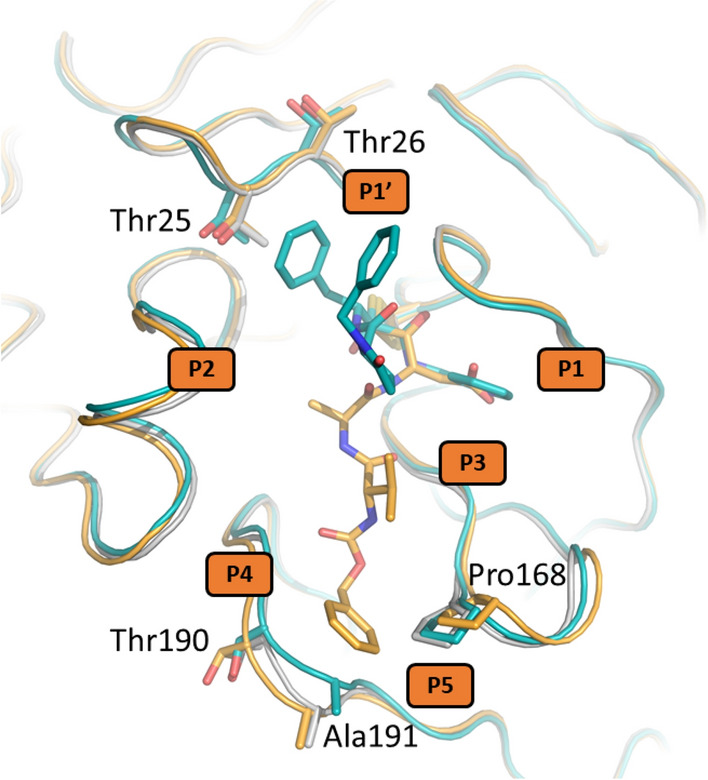
Flexibility induced in the active site of SARS-CoV-2 M^pro^ by compounds **1** and **5** from the ReFRAME library. Grey–Ligand-free M^pro^ (pdb 5r8t), teal–M^pro^ in complex with **1**, orange–M^pro^ in complex with **5**. Figure generated with PyMOL, The PyMOL Molecular Graphics System, Version 1.8.0.5 Schrödinger, LLC (https://pymol.org/2/).

Active site plasticity is important in accommodation of the inhibitors. The P1′ pocket expands on binding of **1**, with the alpha carbons of Thr25 and Thr26 shifting by 0.9 Å and 0.7 Å respectively. Similarly, the P5 pocket expands upon binding to **5**, with Pro168 and Thr190 moving by 1.3 Å and 0.8 Å respectively. Additionally, there is a 1.9 Å shift between Ala191 in the **1** and **5** complexes. In both cases plasticity in response to ligand binding is also observed for the P2 pocket^35^ (Fig. 6).

The pyridine of **1** and the aspartate of **5** extend into the P1 pocket and interact with His163 (Fig. 5c,d). Modelling suggests that the cyclopropyl ring sidechain of **2** is unable to make this interaction and as a consequence does not bind to M^pro^. Similarly, the acid of the aspartate in **5** is in close proximity to Glu166 residue which may cause a charge clash explaining the loss in potency of **5** compared to **4**.

### Prospects for molecular design

Combining the information from the M^pro^ structures of **1** and **5** could be the starting point to design more potent, drug-like inhibitors. As inhibition of Caspase 1 would inhibit inflammation via suppression of the IL-1β, this could provide additional clinical benefit in the treatment of COVID-19^36,37^. Thus inhibitors which possess dual anti-inflammatory and antiviral properties may be desirable. A docking analysis^38,39^of the binding pose of **5** in M^pro^ and the caspase-1 active site reveals multiple shared interactions, indicating that further, more drug-like molecules could be developed which share the potential dual anti-viral/anti-inflammatory polypharmacology of SDZ 224015 (Supplementary Fig. 9).

For PL^pro^, the 4-anilinoquinazoline core is one of the most common scaffolds for generation of tyrosine kinase inhibitors. Thus, it should be possible to rapidly expand from **8** and **9**, to discover new, potentially more potent PL^pro^ inhibitors with the potential to remove kinase activity all together, whilst retaining drug-like properties.

## Discussion

The emergence of COVID-19 has emphasised the need for multiple approaches to tackle viral infections. To bridge the gap between the need to rapidly address a new disease and the time required to safely develop an entirely new medicine, repurposing existing drugs is an attractive alternative^40^.

There have been several clinical efforts to assess the benefits of existing drugs for COVID-19 treatment. Trials have broadly focused on repurposed anti-viral drugs to reduce infection such as remdesivir, as well as the use of existing anti-inflammatory and immunosuppressant compounds to help the body better manage its subsequent response to the infection. Virtual screening has also been used^23,35^, but has only identified boceprevir, an HCV protease inhibitor, as an M^pro^ inhibitor^35^. This molecule was identified in our screen but has disappointing activity (IC_50_ of 3 µM) (Supplementary Table 1).

By contrast, the results described here identify highly potent inhibitors of M^pro^ and for the first time a potent inhibitor of PL^pro^ with drug-like properties. This is also the first description of a non-antiviral molecule to show repurposed PL^pro^ activity. Neither of the two M^pro^ inhibitors discovered in this study were proposed by docking efforts. **4** is a suicide inhibitor which uses a complex mechanism that is difficult to predict^41^. Further, the conformation of **1** within the P1’ pocket of M^pro^ which is driven by intramolecular *pi-pi* stacking (Fig. 3c) is unusual and not readily predicted by in silico approaches^42^.

A recent crystallographic screen of 5000 compounds discovered several compounds which crystallised with M^pro^^43^. One such hit was the EGFR inhibitor pelitinib but disappointingly it only subsequently shows micromolar activity in a cellular screen and was not determined to display significant biochemical inhibition of M^pro^ in this study. In contrast to the M^pro^ crystallographic screen which was also restricted to a single structural form, we were able to study both M^pro^ and PL^pro^ in solution where multiple conformations and oligiomeric forms are present. Pelitinib is compound **10** in our study, but our results show that the 4-aminoquinazoline class of EGFR inhibitors **8** and **9** are more promising; and importantly operate as potent PL^pro^, rather than M^pro^, inhibitors.

By employing optimised screens, we specifically interrogated the two essential SARS-CoV-2 viral proteases, discovering compounds not identified in previous phenotypic screens despite possessing anti-viral activity. The ReFRAME collection has been screened in phenotypic viral-replication^44^ assays. In spite of counter screens, without deconvolution, the results from phenotypic screens can artificially prioritise highly potent compounds such as transcription inhibitors and cytotoxic compounds that have undesirable mechanisms of action precluding therapeutic development^45^ along with undervaluing the potency of viable compounds. Compounds that require revised assay protocols to observe activity, such as **4**, were therefore not previously identified.

In vitro viral replication assays are of limited value for predicting the in vivo pharmacodynamics of candidate molecules^46^ where multi-day assays often underestimate the true in vivo potency. For compounds such as **4**, where a confounding factor is the aqueous stability of the molecule, in vitro data serve to support the mechanism rather than predict in vivo efficacy, where administration frequency, route and immune clearance would positively influence potency^47,48^.

Similarly, potencies of anti-viral activity can vary drastically depending on the methods used. A recent study of the PL^pro^ tool inhibitor GRL-0617^8^ saw potency vary by two orders of magnitude between biochemical (IC_50_ of 2 µM), cytopathic-effect (30 µM), viral RNA detection (> 50 µM) and FFU (IC_50_ > 100 µM) assays. Consequently, there is a prospect that the potencies of both **4** and **8** in vivo may be better than implied by the cytopathic-effect antiviral measure used in this work.

There have been three widespread outbreaks of fatal novel respiratory coronavirus mediated disease in the last two decades^49^. Retrospectively, the dangers of further outbreaks were evident following the first^50^. To avoid the debilitating effects of future coronavirus pandemics or even escape from immune protection, a range of treatments are necessary in which effective antiviral drugs will be a critical component. Protease inhibitors have been highly successful in combating other viral infections^51^. The high conservation of M^pro^ and PL^pro^ between the three strains of coronavirus which cause greatest impact on human health suggest that these are excellent target opportunities for developing small-molecule anti-viral therapeutics.

Anti-viral efforts aim to treat patients who are already infected and halt progression to severe disease^6^. This serves to reduce the burden of disease on fragile health care systems, but must also be employed alongside vaccination^3^ and containment efforts^52^. Vaccination and containment serve to prevent the potential for infection, whereas anti-viral aim to treat those already infected. To be truly useful anti-viral medicines must be broad spectrum and stockpiled prior to an outbreak as suggested for influenza^53^.

Our studies describe the discovery of potent, drug-like inhibitors for both M^pro^ and PL^pro^. These inhibitors display in vitro antiviral activity and have already been shown to be safe for clinical investigation for other therapeutic areas. Given their existing preclinical safety profiles these compounds have the potential for rapid progression towards a clinical setting.

## Methods

### Materials

The ReFRAME library was received from Calibr, Scripps Research, as compounds dissolved to 10 mM in DMSO, spotted in 30 nL volumes in black 384 well plates. All peptides used were prepared with C-terminal amides from Cambridge Research Biochemicals (Billingham, UK) and provided at > 95% purity. Cambridge Research Biochemicals (Billingham, UK) synthesized the ester and acid forms of SDZ-224015 (compounds **4** & **5**) used in follow-up studies, provided at > 95% purity. Additional compound **4** was synthesised as described below. pelitinib (**10**), afatinib (**11**), dacomitinib (**12**) and gefitinib (**13**) were obtained from Tocris (Bristol, UK). The active form of Tarloxotinib (**9**) was from Molport (Riga, Latvia). Compound **3** was from Santa Cruz Biotechnology (Dallas, Texas, USA), Compounds **2**,** 7** and** 8** were from MedChem Express (Sweden); Compound **6** was from Bachem (Bubendorf, Switzerland).

The African monkey kidney cell line Vero E6-GFP was a gift kindly provided by M. van Loock, Janssen Pharmaceutica, Beerse, Belgium.

The hepatocellular carcinoma cell line Huh7 was a gift kindly provided by Ralf Bartenschlager, University of Heidelberg, Germany.

All compounds were obtained at a manufacturer specification of > 98% purity.

Unless otherwise stated all other reagents were from Sigma Aldrich (Poole, UK).

### Construct design and construction

The M^pro^ coding sequence was codon optimised for expression in *E. coli* and synthesised by Integrated DNA technologies (IDT). The M^pro^ expression construct used for crystallization comprises an N-terminal GST region, an M^pro^ autocleavage site, the M^pro^ coding sequence, a hybrid cleavage site recognizable by 3C HRV protease and a C-terminal 6-Histidine tag^54^. The overall construct was flanked by In-Fusion compatible ends for insertion into BamHI-XhoI cleaved pGEX-6P-1 (Sigma). An additional M^pro^ construct was generated with an extended 10-Histidine tag, for enhanced binding to the sensor surface in SPR assays. This construct was amplified by PCR from the above version, with the C-terminal primer incorporating a further 4-Histidines. The resulting amplicon was then inserted into BamHI-XhoI cleaved pGEX-6P-1 by In-Fusion cloning.

The PL^pro^ expression construct was similarly optimised and synthesised and comprised an N-terminal 10 Histidine tag followed by the PL^pro^ sequence (Nsp3 region E746-K1060). This was then directly inserted into NcoI-HindIII digested pOPINF via In-Fusion compatible ends. pOPINF was a gift from Ray Owens (University of Oxford)^55^ (Addgene plasmid # 26042 ; http://n2t.net/addgene:26042 ; RRID:Addgene_26042).

### Protein expression of M^pro^ with authentic termini

The plasmids were used to transform a competent *E. coli* expression cell line based on BL21(DE3)-R3-pRARE. The cells were plated on LB-agar plates containing 50 µg/ml carbenicillin and incubated overnight at 37 °C. The next day multiple colonies were picked and use to inoculate a series of consecutive starter cultures (LB, 50 µg/ml Carbenicillin) of 1 ml, 10 ml and 100 ml. At each stage the culture was grown to the exponential phase (OD_600_ 0.6–2, 200 rpm, 37 °C) before using the total volume of culture to inoculate the next, where the inoculate comprised 10% of the volume of the next culture in the series. Once 100 ml of exponential culture was achieved, 10 ml of this was used to inoculate 1 L of Auto Induction medium (Formedium, Terrific broth base including trace elements, prepared to manufacturer’s instructions with addition of 10 ml glycerol and 50 µg/ml carbenicillin). Cultures were grown for 5 h at 37 °C, 200 rpm, followed by 15–20 h at 18 °C, 200 rpm. Cells were harvested by centrifugation and stored at -80 °C.

### Protein purification of M^pro^ with authentic termini for crystallographic analysis

Cells were resuspended in lysis buffer, 50 mM Tris pH 8, 300 mM NaCl, 10 mM imidazole, 0.03 μg/ml Benzonase, and lysed using an Emulsiflex homogeniser (3 passes, 30 kpsi, 4 °C). Insoluble material was removed by centrifugation (50,000 g, 4 °C). Tagged M^pro^ protein was captured using Nickel-NTA (Takara His60 Superflow Resin) washed with 50 mM Tris pH 8, 300 mM NaCl, 25 mM imidazole, and eluted with 50 mM Tris pH 8, 300 mM NaCl, 500 mM imidazole. To remove the M^pro^ poly-histidine tag, N-terminal His tagged HRV 3C protease was added to the eluted M^pro^ fractions at a ratio of 1 mg 3C protease: 10 mg M^pro^. The mixture was dialysed overnight into 50 mM Tris pH 8, 300 mM NaCl, 0.5 mM TCEP at 4 °C and purified by reverse Nickel-NTA. Gel filtration was performed using a 16/600 Superdex S200 pg column (GE Healthcare) equilibrated in 50 mM Tris pH 8, 300 mM NaCl buffer. M^pro^ was concentrated to 36 mg/ml using a centrifugal filter device with a 10 kDa molecular weight cut off prior to flash freezing using liquid nitrogen.

### Expression and purification of M^pro^-His10

M^pro^-His10 was prepared as for M^pro^ with authentic termini with the following modifications. The HRV 3C protease cleavage and reverse Ni–NTA steps were omitted. Instead, the Ni–NTA purified tagged M^pro^ was dialysed into 50 mM Tris pH 8.5, 100 mM NaCl, 0.5 mM TCEP at 4 °C overnight. The dialysed sample was rapidly diluted with 50 mM Tris pH 8.5 buffer to achieve a final NaCl concentration of 25 mM. The protein was purified by anion exchange chromatography using a 5 ml HiTrap Q HP column (GE Healthcare) on a NaCl concentration gradient between 25 mM to 0.5 M NaCl. Ion exchange chromatography was followed by a gel filtration purification step as described above.

### Expression and purification of cleaved PL^pro^

Cleaved PL^pro^ was prepared as for M^pro^ with authentic termini with the following modifications. A tunable T7 expression strain based on Lemo21 (DE3) was utilised in the expression of PLpro PL^pro^ and 34 μg/ml chloramphenicol was added to all solid and liquid media to maintain its pLemo plasmid. 2 mM Rhamnose was included in the medium in the overnight and sub-culture stages. 0.5 mM Rhamnose was included in the auto induction medium. Instead of the HRV 3C protease, TEV protease was used to cleave the 10 Histidine tag of PL^pro^ at the same mass ratio as described above. A 16/600 Superdex S75 pg column (GE Healthcare) was used for gel filtration chromatography.

### Expression and purification of His10-PL^pro^

His10-PL^pro^ was prepared as for cleaved PL^pro^ with the following modifications. The TEV protease cleavage and reverse Ni–NTA steps were omitted. Instead, the Ni–NTA purified tagged PL^pro^ was dialysed into 50 mM Tris pH 8.8, 100 mM NaCl, 0.5 mM TCEP at 4 °C overnight. The dialysed sample was rapidly diluted with 50 mM Tris pH 8.8 buffer to achieve a final NaCl concentration of 25 mM. The protein was purified by anion exchange using a 5 ml HiTrap Q HP column (GE Healthcare) with a NaCl concentration gradient between 25 mM to 0.5 M NaCl. The ion exchange chromatography was followed by a gel filtration purification step as described above.

### M^pro^ characterisation

The M^pro^ substrate, [5-TAMRA]-AVLQSGFR-[Lys(BHQ-2)]-K-amide was disolved in DMSO (10 mM). This solution was diluted in buffer (150 mM NaCl, 50 mM Tris, 1 mM EDTA, 0.5 mM TCEP, 0.05% (v/v) Triton X-100, pH 7.6), then mixed with M^pro^ solution, to give a final substrate concentration range of 0.8–100 µM and M^pro^ concentration range of 0.3–300 nM. Initial rates were measured using a Pherastar FSX plate reader equipped with a TAMRA filter set.

### M^pro^ inhibition assays

Compounds were received for ReFRAME screening at 10 mM in DMSO at 30 nL volumes in black 384 wells plates; otherwise, dilutions were performed in DMSO from 10 mM stock solutions; 300 nL was transferred to a black 384 well plate using a Mosquito liquid handler (SPT Labtech, Melbourne, UK). 10 µL of the Assay Buffer (150 mM NaCl, 50 mM Tris, 1 mM EDTA, 0.5 mM TCEP, 0.05% (v/v) Triton X-100, pH 7.6) was added to the assay plates to solubilise compounds. 10 µL M^pro^, 90 nM in Assay Buffer, was added to the appropriate wells and incubated with the compounds for 60 min at room temperature. The reaction was started via the addition of 10 µL of the substrate [5-TAMRA]-AVLQSGFR-[Lys(BHQ-2)]-K-amide (where BHQ is Black hole quencher 2), 18 µM in assay buffer. This resulted in final assay conditions of 30 nM M^pro^ and 6 µM substrate, with either 0.1% or 1% DMSO (v/v). The plates were incubated for a further 60 min at room temperature; assays employed a Pherastar FSX plate reader (BMG Labtech, Aylesbury, UK) using a TAMRA filter set. Assay quality was established using Z’, where a low control consisted for substrate alone with balanced DMSO, and the high control was enzyme and substrate without inhibitors but balanced DMSO. The raw fluorescence data was converted to percent inhibition using the same controls as for Z’.

### M^pro^ SPR assay

Cytiva Biacore S200 and T200 machines were used for all SPR experiments. Data were collected at a constant temperature of 20 °C. M^pro^-10His was captured on an NTA chip using standard protocols in running buffer: 20 mM Hepes (pH 7.5), 150 mM NaCl, 50 µM EDTA, 0.05% (v/v) Tween 20 and either 1 or 3% (v/v) DMSO at ~ 8500 RU. The compounds were screened at concentrations ranging from 23 nM to 50 µM adjusted appropriately for each compound, injecting from the lowest to highest concentrations. Scrubber 2 (Biologic software) was used to process and analyse SPR data. Kinetics were fitted using a 1:1 binding model with local Rmax for each concentration where required. Data for the inhibitors were referenced to those for a blank surface and blank injections to normalize for non-specific binding and drift. A DMSO calibration was run to remove excluded volume effect of binding responses between reference and target surface.

### M^pro^ protein observed mass spectroscopy

Protein MS-analyses were performed as described^56^ using a RapidFire RF 365 high-throughput sampling robot (Agilent) attached to an iFunnel Agilent 6550 accurate mass quadrupole time-of-flight (Q-TOF) mass spectrometer operating in the positive ionization mode with the parameters: capillary voltage (4000 V), nozzle voltage (1000 V), fragmentor voltage (365 V), gas temperature (225 °C), gas flow (13 L/min), sheath gas temperature (350 °C), sheath gas flow (12 L/min). The reaction was initiated either by adding SDZ-224015 (compound **4**; 10 mM in DMSO) to a final concentration of 2.5 µM into reaction buffer (20 mM HEPES, pH 7.5, 50 mM NaCl) containing 2.5 µM M^pro^ or by adding M^pro^ (15 µM in 20 mM HEPES, pH 7.5, 300 mM NaCl) to a final concentration of 1 µM into reaction buffer (20 mM HEPES, pH 7.5, 50 mM NaCl) containing SDZ-224015 (5 µM). A sample from the reaction mixture was directly aspirated under vacuum (0.6 s) and loaded onto a C4 solid phase extraction (SPE) cartridge. After loading, the C4 SPE cartridge was washed with 0.1% (v/v) aqueous formic acid to remove non-volatile buffer salts (5.5 s, 1.5 mL/min) and the protein was then eluted from the SPE cartridge with 0.1% (v/v) aqueous formic acid in 85/15 (v/v) acetonitrile/water into the mass spectrometer (5.5 s, 1.25 mL/min). The SPE was cartridge re-equilibrated with 0.1% (v/v) aqueous formic acid (0.5 s, 1.25 mL/min) and a blank water sample was injected before the next reaction sample was aspirated from the assay mixture. Protein spectra were deconvoluted (mass range: 10–60 kDa, m/z range: 950–1300 Da, mass step: 1 Da) using the MaxEnt1 function in Agilent MassHunter Version 7 (Agilent), normalised, and plotted using Graphpad Prism 5.

### Crystallisation and structure determination of M^pro^ in complex with compounds 1 and 5

M^pro^ was thawed and diluted to 6 mg/ml using 20 mM Hepes pH 7.5, 50 mM NaCl. The ligand of interest was dissolved in DMSO to 10 mM and then diluted into the protein solution to a final concentration of 1 mM. The ligand was then allowed to incubate with the protein for two hours at room temperature prior to dispensing plates. The drop composition was 0.15 µL protein ligand solution, 0.3 µL 11% (v/v) PEG 4 K, 0.1 M MES pH 6.5, and 0.05 µL M^pro^ crystal seed stock. The M^pro^ crystal seed stock was prepared by crushing M^pro^ crystals with a pipette tip, suspending them in 30% PEG 4 K, 5% (v/v) DMSO, 0.1 M MES pH 6.5, and vortexing for 60 s with approximately 10 glass beads (1.0 mm diameter, BioSpec products). Reservoir solution was 11% (v/v) PEG 4 K, 5% (v/v) DMSO, 0.1 M MES pH 6.5. Crystals were grown using the sitting drop vapor diffusion method at 20 °C and appeared within 24 h, reaching full size within 36 h.

### Data collection and structure determination

All diffraction data were collected from crystals cryo-cooled to 100 K at Diamond Light Source. X-ray diffraction data for the M^pro^ compound **1** complex were collected at beamline I04-1 at a wavelength of 0.9126 Å and data for the M^pro^ compound **5** complex were collected at I24 at 0.9999 Å. Data were processed using Dials^57^ via Xia2^58^. The datasets were phased using Molrep^59^ and the M^pro^ apo structure^60^. Ligand restraints were generated using GRADE (Global Phasing Ltd) and AceDRG^61^. Crystal structures were manually rebuilt in Coot^62^ and refined using Refmac^63^ and Buster^64^.

### PL^pro^ enzyme characterisation

50 nM PL^pro^ was incubated with titrations of either Ubiquitin-Rhodamine (RnD Systems, Abingdon, UK) (0.1–10 µM) or [5-TAMRA]- VLRLRGG-[Lys(BHQ-2)]-amide (1–100 µM) in either 150 mM NaCl, 50 mM Tris, 1 mM EDTA, 0.5 mM TCEP, 0.05% (v/v) Triton X-100, pH 7.6 or 800 mM sodium citrate, 200 mM Tris, 0.5 mM TCEP, 0.05% (v/v) Triton X-100, pH 7.6. Initial rate was established using a Pherastar FSX plate reader using either a FITC (Ubiquti-Rhodamine) or a TARMA ([5-TAMRA]- VLRLRGG-[Lys(BHQ-2)]-amide) filter set.

### PL^pro^ biochemical assay

Compound plates were prepared as for the M^pro^ biochemical assay. 10 µL assay buffer (800 mM sodium citrate, 200 mM Tris, 0.5 mM TCEP, 0.05% (v/v) Triton X-100, pH 7.6) was added to the assay plates to solubilise the compound. 10 µL of PL^pro^ at 75 nM in assay buffer, was added to the appropriate wells and incubated with the compounds for 60 min at room temperature. The reaction was started via the addition of 10 µL of the substrate [5-TAMRA]- VLRLRGG-[Lys(BHQ-2)]-amide at 6 µM in assay buffer. This resulted in final assay conditions of 25 nM PL^pro^ and 2 µM substrate, with either 0.1% or 1% DMSO (v/v). The plates were incubated for a further 60 min at room temperature and the assay was read on a Pherastar FSX plate reader (BMG Labtech, Aylesbury, UK) using a TAMRA filter set. Assay quality was established as for the M^pro^ biochemical assay. Data were converted to percent inhibition in the same manner as the M^pro^ assay.

### PL^pro^ kinetic assays

Assays employed the same conditions as the PL^pro^ inhibition assays, except the compound and substrate were prepared in the assay plate and the assay was started with an injection of 10 µL of enzyme using a Pherastar FSX plate reader. Fluorescence was measured every 15 s post injection using a TAMRA filter set for 60 min. Data were zeroed to the date measured at time of injection and fit to a two-state inhibition model.

### Effect of hoffmeister salt concentration on PL^pro^ activity

[5-TAMRA]- VLRLRGG-[Lys(BHQ-2)]-amide was dissolved in buffers with increasing concentration of sodium phosphate (0.15–1.5 M sodium phosphate, 1 mM EDTA, 0.5 mM TCEP, 0.05% Triton-X100, pH 7.4). This was then mixed with PL^pro^ in a matched buffer to give final conditions of: 2 µM substrate and 30 nM enzyme. The initial rate was established using a Pherastar FSX plate reader using either a FITC (Ubiqutin-Rhodamine) or a TARMA ([5-TAMRA]-VLRLRGG-[Lys(BHQ-2)]-amide) filter set.

### Effect of Hoffmeister salt concentration on tarloxotinib bromide inhibition

This assay was performed in the same manner as the PL^pro^ biochemical assay, except the buffer was either 0.15, 0.75, 1 or 1.5 M sodium phosphate, 1 mM EDTA, 0.5 mM TCEP, 0.05% Triton-X100 pH7.4.

### Cell culture

Huh-7 cells stably expressing H2B-mCherry were generated using lentiviral vectors containing a CMV-H2B-mCherry-P2A-BlastR cassette. The Huh-7 mCherry cells were maintained in Dulbecco’s modified Eagle’s medium (DMEM; Gibco) supplemented with 10% v/v fetal calf serum (FCS; Biowest), 10 ml HEPES, 5 ml NEAA, and 1 × Pen-strep (Gibco) and kept under 5% CO2 on 37 °C. Assay medium contained only 4% FCS.

### Virus culture

SARS-CoV-2 strain BetaCov/Belgium/GHB-03021/2020 recovered from a nasopharyngeal swab taken from an asymptomatic patient returning from Wuhan, China at the beginning of February 2020 was sequenced on a MinION platform (Oxford Nanopore). After serial passaging on Huh7 and Vero E6 cells, infectious content of the virus stock was determined by titration on HUH7 cells using the Spearman-Kärber method. All virus-related work was carried out in certified, high-containment biosafety level-3 facilities of KU Leuven Rega institute.

### Antiviral assay

To measure inhibition of the SARS-CoV-2 cytopathic effect, 96-well plates (Corning 3300) were plated with HUH7_mCherry cells at 6000 cells/well in 100 µl. The day after (Day 0), compound was added in a dilution series for concentration response studies. After two hours, addition of virus dilution (final MOI 0.004) was performed and plates were left for incubation at 37 °C, 5% CO2 for four days.

Cytotoxicity was assessed in parallel using the same protocol, albeit without the addition of virus dilution. Plates were imaged on an Arrayscan XTI, Thermofisher.

### Image acquisition and analysis

At day four post-infection, mCherry signal was captured using wide field fluorescence imaging by exciting at 560_25 nm and emitting with the BGRFRN filter set. A 5 X objective sufficed to capture 65–70% of an entire well on a 96well plate (4 pictures in total). The optimal exposure time was determined based on fluorescence intensity and was set on 0.09 s. A 2 × 2 binning was used and autofocus plane count was reduced to increase image acquisition speed. An image analysis protocol was developed in-house by using the SpotDetector bioapplication (Cellomics, Thermofisher). After background reduction on the raw image files, a fixed fluorescent intensity threshold was determined for the identification of mCherry cells. Afterwards, the number of fluorescent cells (‘object count’) was calculated per well and compared to the positive (cell control) and negative (virus) control.

### Synthesis of SDZ-224015 (compound 4)

Commercially-sourced reagents (Sigma-Aldrich, Inc.; Fluorochem Ltd; Bachem AG) were used as received. Reactions were performed in anhydrous solvents (Sigma-Aldrich Inc.). Purifications, reaction work-ups, and extractions were performed using HPLC grade solvents (Sigma-Aldrich Inc.). A Stuart SMP-40 automated melting point apparatus was used to determine melting points (MP). A Bruker Tensor-27 Fourier transform infrared spectrometer was used for infrared (IR) spectroscopy. A Unipol (Schmidt Haensch) polarimeter was used for optical rotation (α) measurements. A Thermo Scientific Exactive mass spectrometer (ThermoFisher Scientific) operated in the positive ionization mode was employed for high-resolution mass spectrometry (HRMS) using electrospray ionization (ESI) mass spectrometry (MS); data are presented as a mass-to-charge ratio (m/z). A Bruker AVANCE AVIIIHD 600 spectrometer equipped with a 5 mm BB-F/^1^H Prodigy N_2_ cryoprobe was used for nuclear magnetic resonance (NMR) spectroscopy. Proton chemical shifts are reported in parts per million (ppm) downfield from tetramethylsilane, the residual protium in the NMR solvent is used as a reference (DMSO-*d*_*6*_: δ = 2.49 ppm). Carbon chemical shifts are reported in parts per million (ppm) in the scale relative to the NMR solvent (DMSO-*d*_*6*_: δ = 39.52 ppm). NMR data are reported as: chemical shift, multiplicity (m: multiplet, s: singlet, d: doublet, dd: doublet of doublets, t: triplet, q: quartet), coupling constant (*J*, Hz), and integration.

Ethyl (5*S*,8*S*,11*S*)-11-(2-((2,6-dichlorobenzoyl)oxy)acetyl)-5-isopropyl-8-methyl-3,6,9-trioxo-1-phenyl-2-oxa-4,7,10-triazatridecan-13-oate (SDZ-224015, Z-VAD-DCB, compound **4**) was synthesized from ethyl (*S*)-3-((*R*)-2,2-dimethyl-1,3-dioxolan-4-yl)-3-(phenylamino)propanoate^65^ and Z-Val-Ala-OH in five steps as reported^66^. However, the final oxidation reaction of the reported synthesis of **4**^66^ was modified, due to the insolubility of the starting material in pure dichloromethane, the optimised protocol is given below:

A solution of ethyl (5*S*,8*S*,11*S*)-11-((*R*)-2-((2,6-dichlorobenzoyl)oxy)-1-hydroxyethyl)-5-isopropyl-8-methyl-3,6,9-trioxo-1-phenyl-2-oxa-4,7,10-triazatridecan-13-oate^65^ (1.4 g, 2.0 mmol, 1.0 equiv.) in DMSO (3.0 mL) was diluted with dichloromethane (40 mL). To the resulting clear solution, Dess-Martin periodinane^67^ (3.39 g, 8.0 mmol, 4.0 equiv.) was added at 0° C under ambient atmosphere; the reaction mixture was stirred at 0 °C for 2 h, then for 2 h at ambient temperature, before aqueous phosphate buffer (100 mL, 0.1 M, pH 7) containing sodium metabisulfite (6 g) was added at 0 °C. The resulting mixture was vigorously stirred at ambient temperature for 30 min, then five times extracted with dichloromethane. The combined organic extracts were washed with saturated aqueous NaHCO_3_ solution, dried over anhydrous Na_2_SO_4_, filtered, evaporated; the residue was then purified by reverse phase HPLC (20 mL/min; linear gradient over 39 min: 2% → 98% acetonitrile in water, each containing 0.1% (v/v) formic acid; t_R_ = 27.0 min) using a Shimadzu HPLC purification system (composed of DGU-20A, 2 LC-20AR, CBM-20A, SPD-20A, and FRC-10A units) equipped with a C18 Grace VYDAC 218TP101522 column (Grace Davison Discovery Sciences) to afford 199 mg (15%) of purified SDZ-224015 (compound **4**). The analytical data are in agreement with those reported^66^. White solid, m.p.: 178–180 °C; ^1^H NMR (600 MHz, 300 K, DMSO-*d*_*6*_): δ = 8.62 (d, *J* = 7.5 Hz, 1H), 8.12 (d, *J* = 6.6 Hz, 1H), 7.60 − 7.59 (m, 2H), 7.55 (dd, *J* = 9.2, 6.9 Hz, 1H), 7.36 − 7.34 (m, 4H), 7.32 − 7.29 (m, 1H), 7.26 (d, *J* = 8.7 Hz, 1H), 5.18 (d, *J* = 17.2 Hz, 1H), 5.14 (d, *J* = 16.4 Hz, 1H), 5.03 (d, *J* = 12.7 Hz, 1H), 5.00 (d,* J* = 12.7 Hz, 1H), 4.64 (q, *J* = 6.8 Hz, 1H), 4.27 (app. pent., *J* = 7.2 Hz, 1H), 4.05 (q, *J* = 7.1 Hz, 2H), 3.87 (dd, *J* = 7.9, 7.4 Hz, 1H), 2.85 (dd, *J* = 16.5, 5.8 Hz, 1H), 2.65 (dd, *J* = 16.5, 7.2 Hz, 1H), 1.98 − 1.92 (m, 1H), 1.22 (d, *J* = 7.0 Hz, 3H), 1.16 (t, *J* = 7.1 Hz, 3H), 0.85 (d, *J* = 6.8 Hz, 3H), 0.81 ppm (d, *J* = 6.7 Hz, 3H); ^13^C NMR (150 MHz, 300 K, DMSO-*d*_*6*_): δ = 199.5, 172.9, 171.0, 170.1, 163.2, 156.1, 137.0, 132.6, 132.0, 130.8, 128.5, 128.3, 127.7, 127.6, 67.7, 65.4, 60.3, 59.8, 52.8, 48.1, 34.2, 30.3, 19.2, 18.0, 17.7, 13.9 ppm; IR (film): ṽ = 3293, 3067, 2964, 2936, 1733, 1689, 1639, 1538, 1434, 1374, 1287, 1247, 1195, 1148, 1040 cm^–1^; HRMS (ESI): m/z calculated for C_30_H_36_O_9_N_3_Cl_2_ [M + H]^+^: 652.1823, found: 652.1823; ${[\alpha ]}_{D}^{25}$=  − 47.0 (c = 1.0, acetone).

### Hot spot comparison between M^pro^ and caspase 1

Fragment Hotspot Maps were calculated for structures 6YB7 and 1SC4 using the Hotspots API^38,39^. The method uses molecular probes, atomic interaction propensity and a local buriedness measure to highlight key hotspots within the binding site.

### Research ethics statement

All methods were carried out in accordance with relevant guidelines and regulations. All experimental protocols were approved by either, an internal research committee at Exscientia. Ltd, the ReFRAME committee or the CARE consortium. Viral swabs were obtained with prior patient's written informed consent for use in research.

## Supplementary Information

Supplementary Information.

## Data Availability

References pointing the reader towards code used for hotspot mapping are provided in the methods section.
