# Publisher Correction: Bispecific repurposed medicines targeting the viral and immunological arms of COVID-19

**DOI:** 10.1038/s41598-021-99041-1

**Published:** 2021-09-28

**Authors:** Martin A. Redhead, C. David Owen, Lennart Brewitz, Amelia H. Collette, Petra Lukacik, Claire Strain-Damerell, Sean W. Robinson, Patrick M. Collins, Philipp Schäfer, Mark Swindells, Chris J. Radoux, Iva Navratilova Hopkins, Daren Fearon, Alice Douangamath, Frank von Delft, Tika R. Malla, Laura Vangeel, Thomas Vercruysse, Jan Thibaut, Pieter Leyssen, Tu-Trinh Nguyen, Mitchell Hull, Anthony Tumber, David J. Hallett, Christopher J. Schofield, David I. Stuart, Andrew L. Hopkins, Martin A. Walsh

**Affiliations:** 1 Exscientia, The Schrödinger Building, Oxford Science Park, Oxford, OX4 4GE UK; 2 Diamond Light Source Ltd., Harwell Science and Innovation Campus, Didcot, OX11 0DE UK; 3 Research Complex at Harwell, Harwell Science and Innovation Campus, Didcot, OX11 0FA UK; 4 Division of Structural Biology, Wellcome Centre for Human Genetics, University of Oxford, Oxford, OX3 7BN UK; 5 Instruct-ERIC, Oxford House, Parkway Court, John Smith Drive, Oxford, OX4 2JY UK; 6 Department of Chemistry, Chemistry Research Laboratory,, The Ineos Oxford Institute for Antimicrobial Research, 12 Mansfield Road, Oxford, OX1 3TA UK; 7 KU Leuven Department of Microbiology, Immunology and Transplantation, Rega Institute, 3000 Leuven, Belgium; 8 Calibr, Scripps Research, 11119 N Torrey Pines Road, La Jolla, CA 92037 USA; 9 Structural Genomics Consortium, University of Oxford, Old Road Campus, Roosevelt Drive, Headington, OX3 7DQ UK; 10 Department of Biochemistry, University of Johannesburg, Auckland Park, 2006 South Africa

Correction to: *Scientific Reports* 10.1038/s41598-021-92416-4, published online 24 June 2021

The original version of this Article contained errors in Table 1, where the molecules were incorrectly displayed. The original Table 1 and accompanying legend appear below.

**Table 1:** Structure–activity relationship of 4-aminoquinazoline EGFR inhibitors against SARS-CoV-2 PL^pro^.


Compound number	Generic name	R^1^	R^2^	R^3^	R^4^	R^5^	IC_50_ (µM) (range)
**8**	Tarloxotinib	**Br**	**F**	**N**	**N**		0.3 (0.1–0.5)
**9**	Tarloxotinib (ac)	**Br**	**F**	**N**	**N**		0.3 (0.1–0.4)
**10**	Pelitinib	**Cl**	**Cl**				> 100*
**11**	Afatinib	**Cl**	**Cl**	**N**			12 (11–16)
**12**	Dacomitinib	**Cl**	**Cl**	**N**			4 (3–5)
**13**	Gefitinib	**Cl**	**Cl**	**N**			7 (6–11)

*The range could not be established due to lack of potency.

The original Article has been corrected.

