# Supplementary material for: Bispecific repurposed medicines targeting the viral and immunological arms of COVID-19
Source: Sci Rep. 2021 Jun 24;11:13208. doi: 10.1038/s41598-021-92416-4 (PMC8225628; doi:10.1038/s41598-021-92416-4)
Supplement: Supplementary file 1 — Supplementary Information. [file 41598_2021_92416_MOESM1_ESM.docx]

**Supplementary Information**

**Supplementary Table 1. ReFRAME Hits IC50s**

| **Name** | **M^pro^** | | **PL^pro^** | |
| --- | --- | --- | --- | --- |
|  | **IC_50_ (M)*** | **% inhibition at 10 µM** | **IC_50_ (M)*** | **% inhibition at 10 µM** |
| hypericin | 4E-06 | 71 | >5e-6 | 12 |
| Pyrantel pamoate | 4E-06 | 70 | >5e-6 | 29 |
| SENNOSIDES | 7E-07 | 92 | >5e-6 | 72 |
| (+)-CP-85958 | 2E-06 | 70 | >5e-6 | 17 |
| talosalate | 4E-06 | 80 | >5e-6 | 13 |
| Boceprevir | 5E-06 | 62 | >5e-6 | -2 |
| fondaparinux (sodium) | 2E-06 | 80 | >5e-6 | 23 |
| MELARSONYL | 1E-06 | 99 | >5e-6 | 11 |
| SDZ 224015 | 4E-07 | 95 | >5e-6 | 19 |
| oxantel (pamoate) | 4E-06 | 77 | >5e-6 | 11 |
| erythrosine | 4E-06 | 58 | >5e-6 | 20 |
| xanthohumol | >5e-6 | 11 | >5e-6 | 11 |
| L 680833 | >5e-6 | 36 | >5e-6 | 11 |
| amentoflavone | >5e-6 | 34 | >5e-6 | 4 |
| (-)-epicatechin gallate | >5e-6 | 46 | >5e-6 | 11 |
| acetomeroctol | >5e-6 | 79 | >5e-6 | 7 |
| BGC-20-1531 | >5e-6 | 58 | >5e-6 | 24 |
| nifursol | >5e-6 | 43 | >5e-6 | 23 |
| LY 223982 | >5e-6 | 43 | >5e-6 | 22 |
| [(-)-epicatechin] | >5e-6 | 48 | >5e-6 | 16 |
| provecta | >5e-6 | 70 | >5e-6 | 12 |
| micafungin Sodium | >5e-6 | 50 | >5e-6 | 24 |
| ABT-957 | >5e-6 | 73 | >5e-6 | 17 |
| mercuric chloride | >5e-6 | 89 | >5e-6 | 16 |
| susalimod | >5e-6 | 31 | >5e-6 | 22 |
| LM-1453 | >5e-6 | 56 | >5e-6 | 16 |
| SDZ-880-540 | >5e-6 | -1 | >5e-6 | 15 |
| aescin | >5e-6 | 18 | 2E-06 | 63 |
| PMX-30063 | >5e-6 | 11 | 2E-06 | 30 |
| tarloxotinib bromide | >5e-6 | 57 | 4E-07 | 73 |
| taspoglutide | >5e-6 | 16 | 9E-07 | 93 |
| teicoplanin | >5e-6 | 73 | 7E-07 | 88 |
| teicoplanin | >5e-6 | 40 | 2E-06 | 81 |
| piramal | >5e-6 | 9 | 5E-06 | 31 |

*’>5e-6’ indicates the compound was considered not potent enough to determine an IC_50_.

**Supplementary Table 2. Data collection and refinement statistics**

|  | M^pro^ with compound **1** | M^pro^ with compound **5** |
| --- | --- | --- |
| **Data collection** |  |  |
| Wavelength (Å) | 0.9126 | 0.999 |
| Space group | C2 | C2 |
| Cell dimensions |  |  |
| *a*, *b*, *c* (Å) | 113.95 53.08 44.55 | 112.87 52.94 45.53 |
| α, β, γ (°) | 90.00 103.02 90.00 | 90.00 103.05 90.00 |
| Resolution (Å) | 55.51-1.30 (1.32-1.30) | 54.98-1.70 (1.73-1.70) |
| *R*_meas_ | 0.22 (6.4) | 0.25 (3.32) |
| *R*_merge_ | 0.20 (6.0) | 0.12 (2.92) |
| *I* / σ*I* | 10.7 (1.9) | 7.6 (2/3) |
| CC1/2 | 0.996 (0.310) | 0.989 (0.301) |
| Completeness (%) | 97.2 (88.7) | 99.9 (99.8) |
| Redundancy | 10.1 (1.9) | 6.2 (6.0) |
|  |  |  |
| **Refinement** |  |  |
| Resolution (Å) | 47.97-1.30 | 54.98-1.70 |
| No. reflections | 61989 | 24397 |
| *R*_work_ / *R*_free_ | 0.139 / 0.176 | 0.168/0.200 |
| No. atoms |  |  |
| Protein | 2438 | 2386 |
| Ligand/ion | 48 | 51 |
| Water | 302 | 170 |
| *B*-factors |  |  |
| Protein | 14.9 | 18.8 |
| Ligand/ion | 22.7 | 46.5 |
| Water | 29.0 | 26.4 |
| R.m.s. deviations |  |  |
| Bond lengths (Å) | 0.007 | 0.004 |
| Bond angles (°) | 1.394 | 1.252 |
| Ramachandran |  |  |
| Favoured (%) | 98.35 | 97.36 |
| Outliers (%) | 0.33 | 0.33 |

*Values in parentheses are for highest-resolution shell.

**
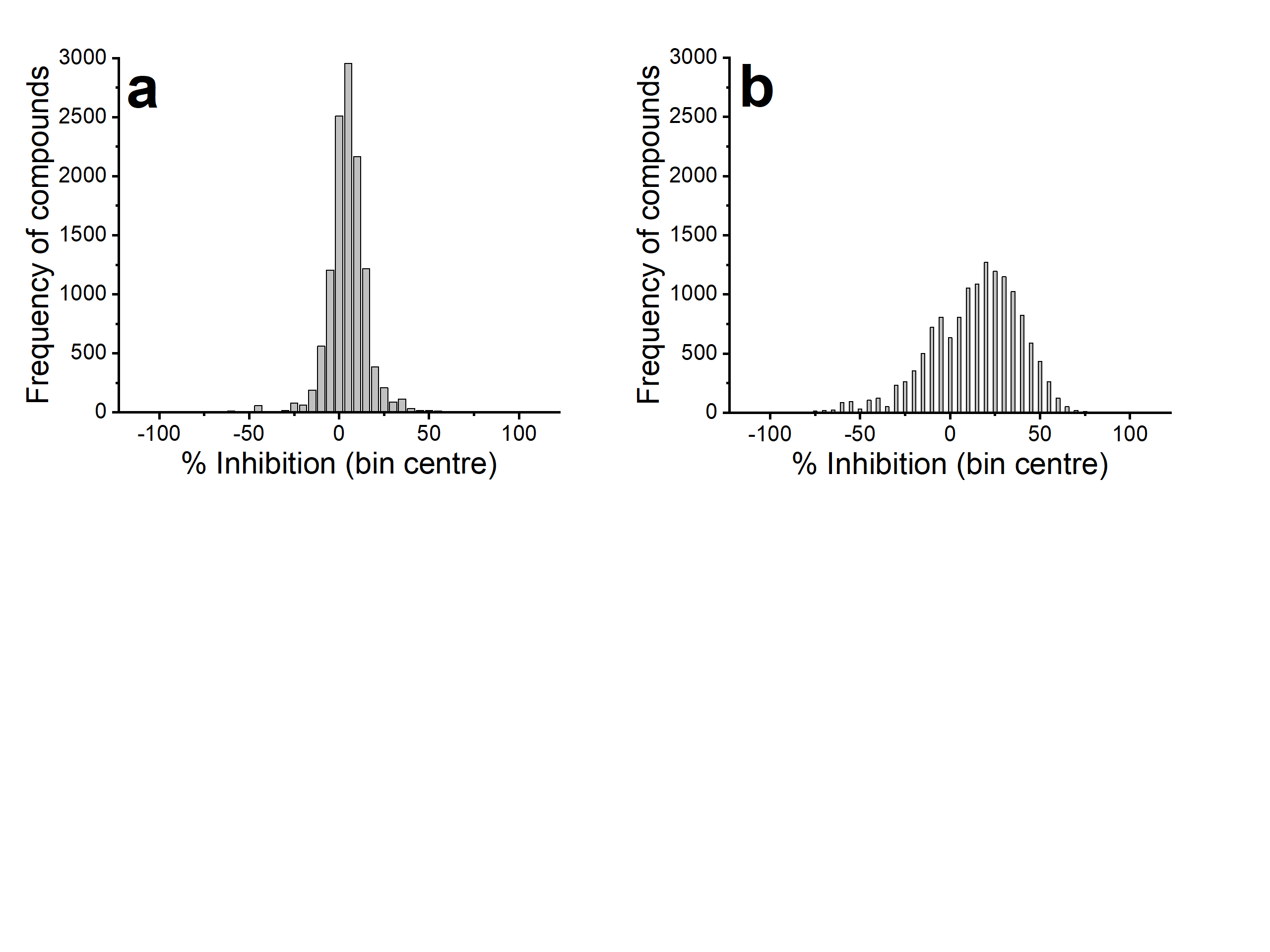
**

**Supplementary Fig. 1. Activity distributions of high through put screens of SARS-CoV-2 M^pro^ and PL^pro^.** The two graphs show histograms of the output of the HTS campaigns using the ReFRAME library against M^pro^ (a) and PL^pro^ (b). The x-axis shows binned inhibition, and the y-axis the frequency of inhibition in each bin.

**
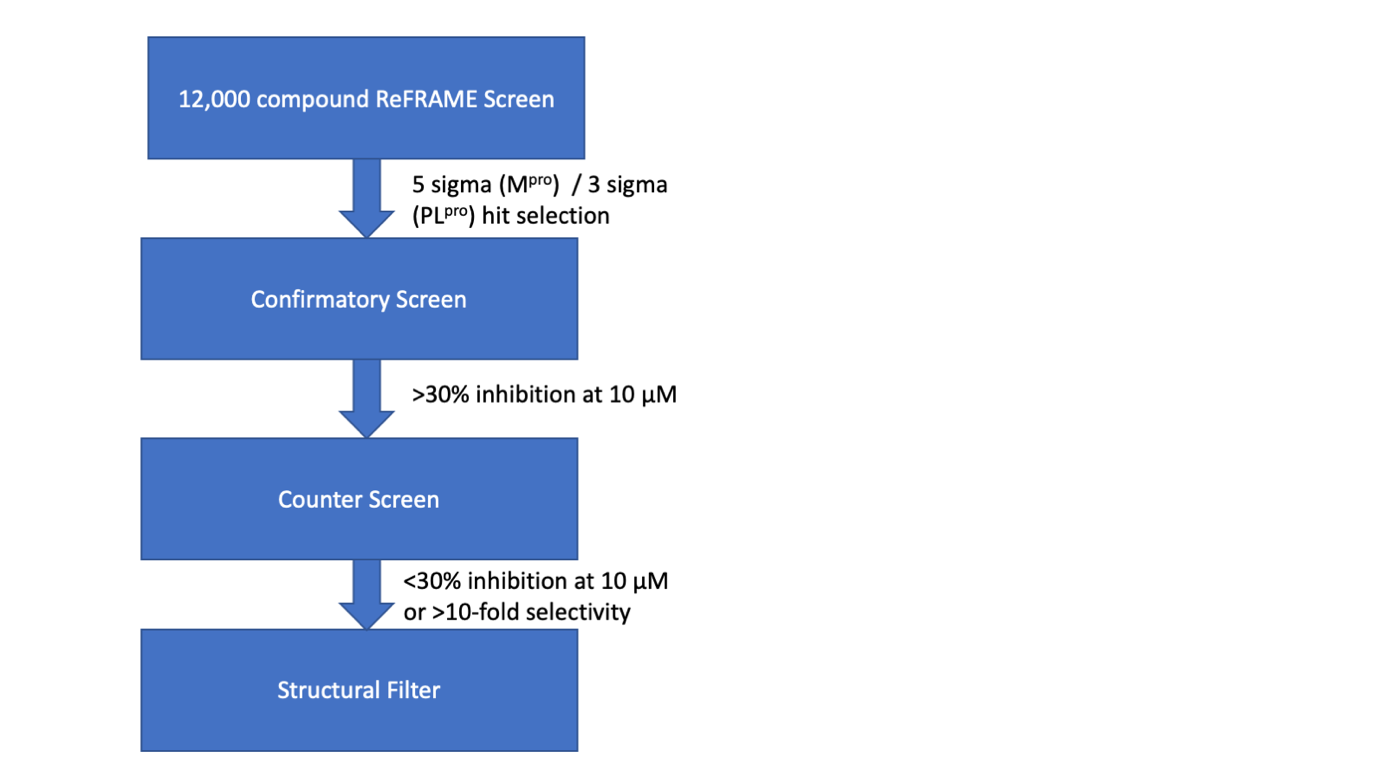
**

**Supplementary Fig. 2.** Screening cascade for the ReFRAME library and selection criteria.


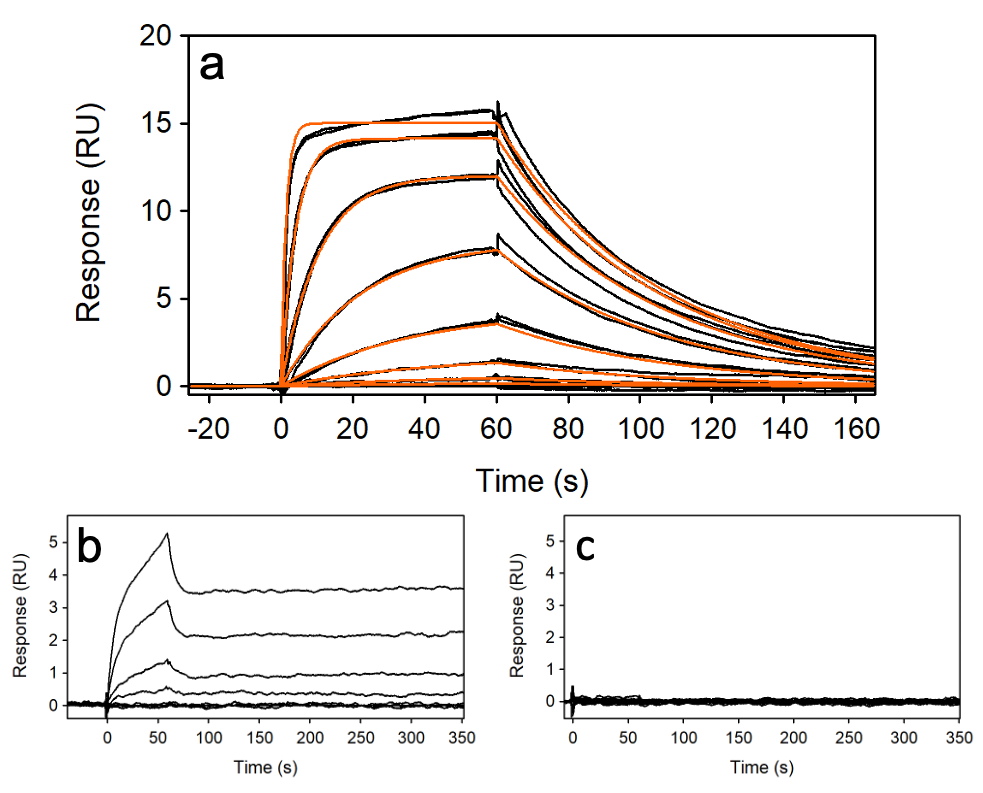


**Supplementary Fig. 3. Binding kinetics of M^pro^ inhibitors.** Panel **a** binding of compound **1** to immobilized M^pro^. Compound is injected at 3-fold concentration series in duplicates (23 nM – 50 µM). Data obtained are show with a black line, with orange lines showing a fit to a 1:1 binding model using full fitting. The fit results determine that compound **1** is binding with kinetic parameters: k_a_=1.40*10^4^ (±0.04) M^-1^s^-1^, k_d_=0.022(±0.06) s^-1^ and K_D_=1.61(±0.03) µM (brackets display fitting error as the standard error of residuals). Panel **b** shows compound **4** binding to immobilized M^pro^ captured at 2500 RU. Compound **4** was injected at 3-fold concentration series from 0.45 nM to 1 µM Panel **c** shows responses of SDZ to M^pro^ in the presence of slow off-rate inhibitor N3 – no binding observed.

Data for compound **4** displays a very slow off-rate and complex dissociation. Due to the nature of the peptide and the binding mechanism we are showing the peptide is binding to the M^pro^, however binding is known to be irreversible. Due to the complex mechanism of suicide inhibition it is not possible to obtain accurate kinetic parameters from this dataset. The compound does not bind to N3 blocked receptor indicating the binding is to the same site on the protein.


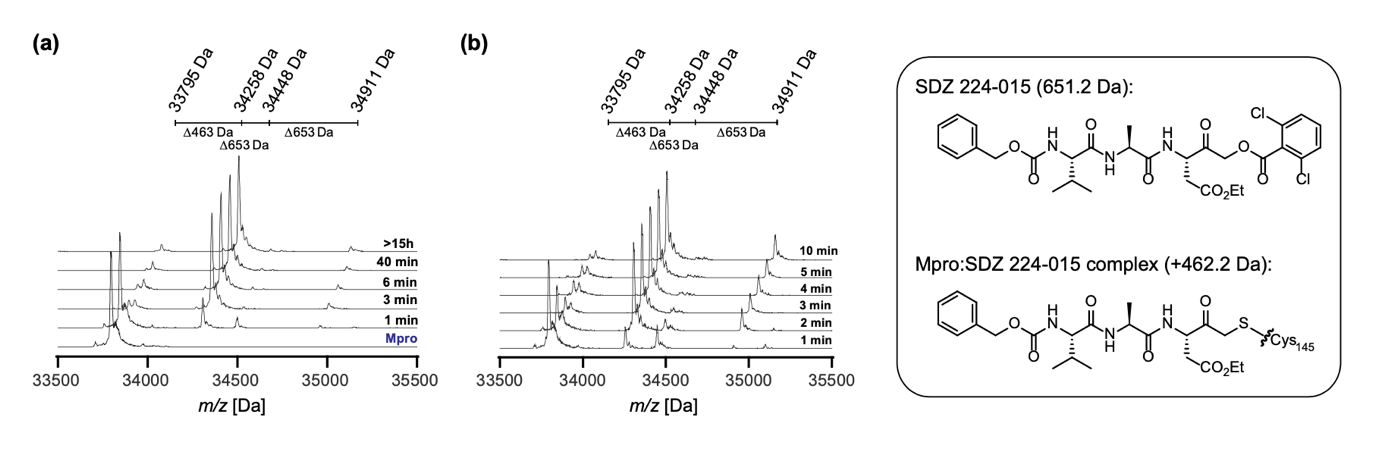


**Supplementary Fig. 4. Protein observed mass spectrum of M^pro^ incubated with compound 4.** Protein observed mass spectrometry shows rapid formation of a 462 Da adduct to M^pro^ in the presence of compound **4**, indicating the loss of the AMOK group.


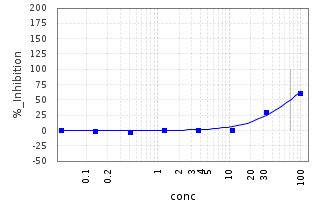

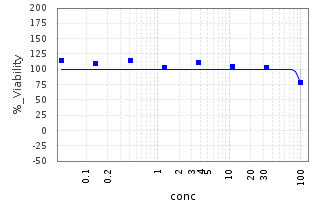


**Supplementary Fig. 5. Antiviral activity of 1 in HUH7_mCherry cells.** The x axis displays concetration in µM and the y axis displays inhibition of viral replication.


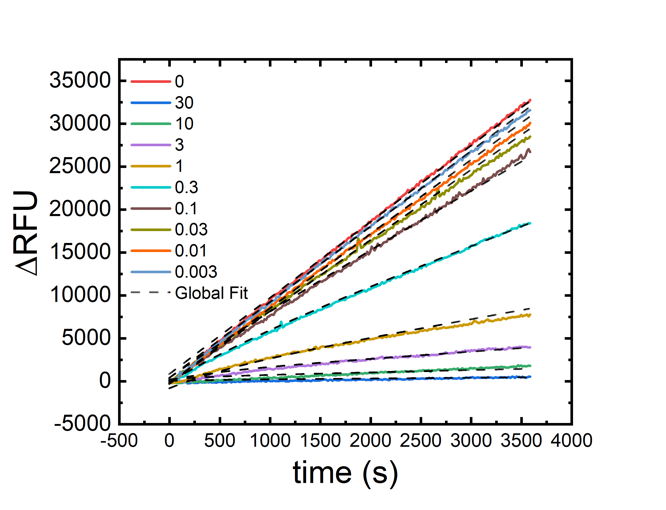

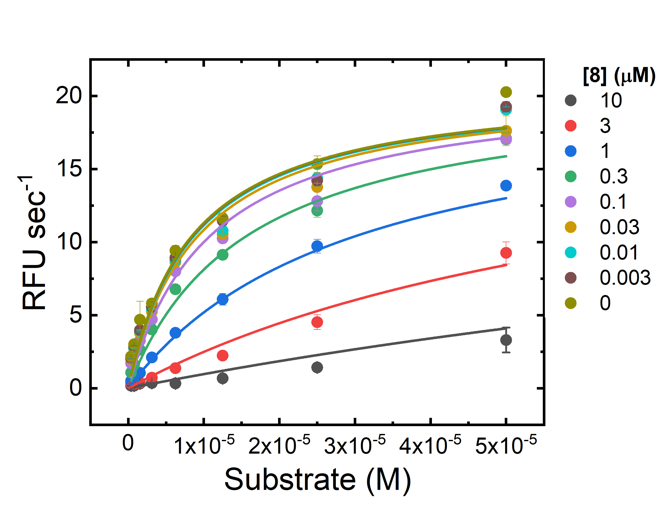


**Supplementary Fig. 6. Time-dependent tarloxotinib inhibition of SARS-CoV-2 PL^pro^ and substrate dependence of inhibition.** The left-hand graph shows increase in fluorescence against time with each curve the average of four replicates (error bars not shown). The coloured lines show the data obtained and the dashed black line shows a global fit to a two-state time dependent inhibition model. Concentration is annotated next to the key in µM. The right hand graph shows a PL^pro^ substrate-velocity graph in increasing concentrations of **8**. Points are the average of two technical repeats, with error bars showing the range, the lines represent a fit to a competitive kinetic model.

**
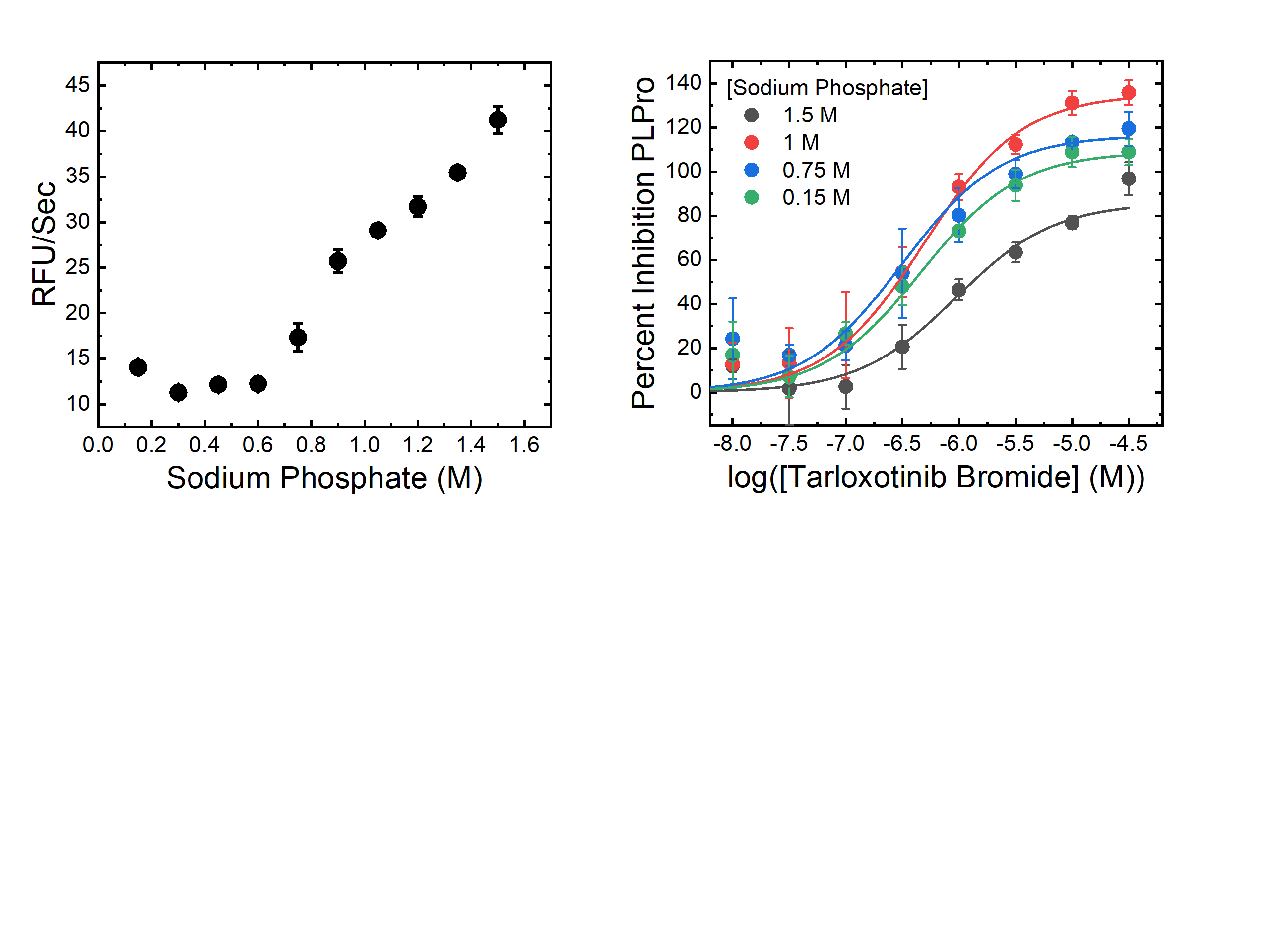
**

**Supplementary Fig. 7. Effect of Hoffmeister salts on PL^pro^ activity**. The left panel shows the effect of increasing concentrations of sodium phosphate on the enzymatic activity of PL^pro^. Data are plotted as the average of four replicates, with error bars showing the standard deviation. The right panel shows the IC_50_ for Tarloxotinib bromide against PL^pro^ at differing concentrations of sodium phosphate. Data are plotted as the average of four replicates with error bars showing the standard deviation, with the fit to a 3 parameter IC50 shown as a solid line.


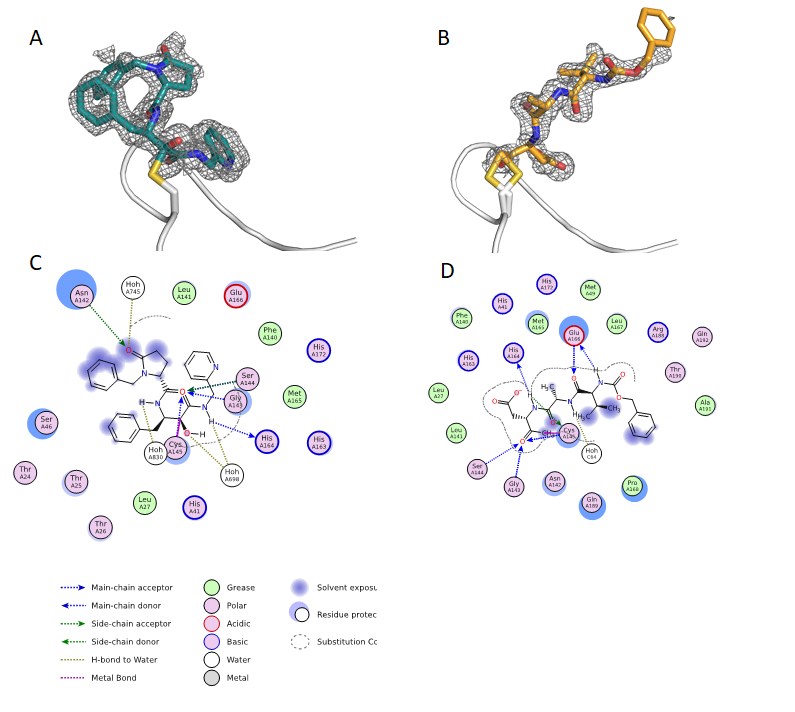


**Supplementary Fig. 8.** Fo-Fc difference map density (grey mesh, contoured at 2*σ*) showing **A** 1 (teal) and **B** 5 (orange) bound covalently to Cys145 of SARS-CoV-2 M^pro^. In **C** and **D** schematics oif the ligand environment are show for compounds 1 and 5 respectively. Panel **A** and **B** generated with PyMOL, The PyMOL Molecular Graphics System, Version 2.0 Schrödinger, LLC (<https://pymol.org/2/>). Panel **C** and **D** generated with WinCoot 0.9.4 (<https://bernhardcl.github.io/coot/>)


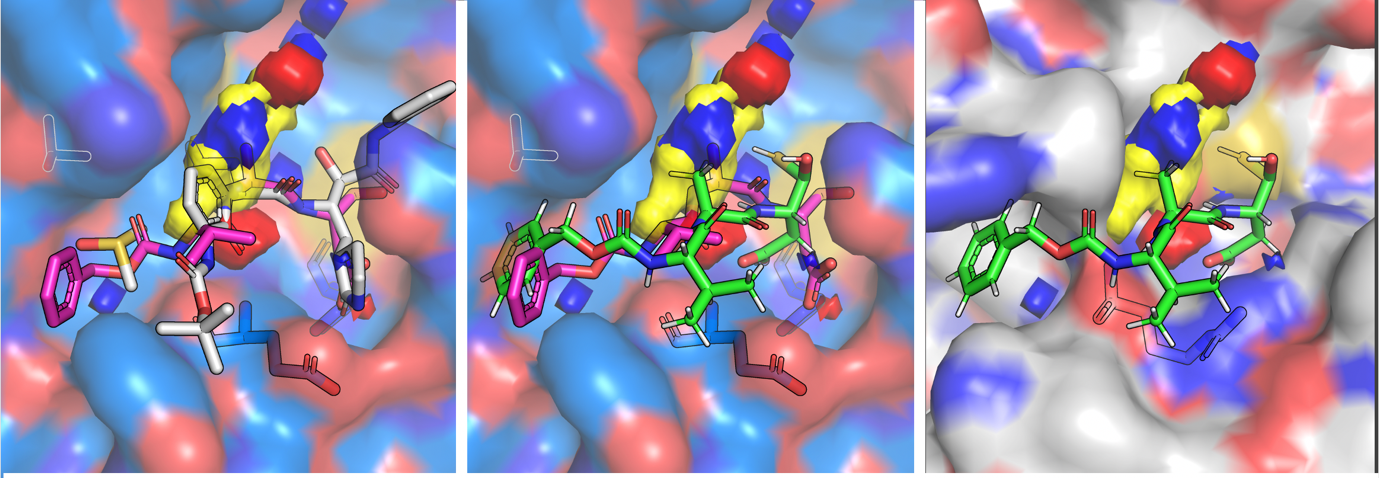
 **Supplementary** **Figure 9. Comparison of compound 5 binding to M^pro^ and Caspase 1.** The Fragment Hotspot Maps were calculated for PDB structure 6Y2F (shown as a blue surface), showing the most productive regions for binding apolar (yellow) donor (blue) and acceptor (red) functional groups. (**a)** Aligned structure of compound **5** (magenta sticks) with the crystal structure of PDB 6Y2F. Both compounds **5** and the N3 inhibitor adopt similar binding poses, and key interactions with his163 in the P1 pocket and glu166 in the P2 pocket are maintained by Compound **5**. (**b**) Shows the docking pose of compound **5** in green, as covalently docked with Caspase 1 (PDB code 1rww), showing a similar conformation to the bound pose in M^pro^. (**c**) The same docked pose in the Caspase 1 binding site, alongside the hotspot maps calculated for M^pro^. The similar locations of key interactions and overall pocket shape relative to the reactive cysteine should facilitate the design of inhibitors targeting both M^pro^ and Caspase 1. Figure generated with PyMOL, The PyMOL Molecular Graphics System, Version 2.0 Schrödinger, LLC (https://pymol.org/2/).
